# Child dietary patterns in *Homo sapiens* evolution: A systematic review

**DOI:** 10.1093/emph/eoac027

**Published:** 2022-07-26

**Authors:** Lora L Iannotti, Emmanuel A Gyimah, Miranda Reid, Melissa Chapnick, Mary Kate Cartmill, Chessa K Lutter, Charles Hilton, Theresa E Gildner, Elizabeth A Quinn

**Affiliations:** Brown School, Institute for Public Health, Washington University in St. Louis, 1 Brookings Drive, Campus Box 1196, St. Louis, MO 63130, USA; Brown School, Institute for Public Health, Washington University in St. Louis, 1 Brookings Drive, Campus Box 1196, St. Louis, MO 63130, USA; Brown School, Institute for Public Health, Washington University in St. Louis, 1 Brookings Drive, Campus Box 1196, St. Louis, MO 63130, USA; Brown School, Institute for Public Health, Washington University in St. Louis, 1 Brookings Drive, Campus Box 1196, St. Louis, MO 63130, USA; Brown School, Institute for Public Health, Washington University in St. Louis, 1 Brookings Drive, Campus Box 1196, St. Louis, MO 63130, USA; RTI International, 701 13th St NW #750, Washington, DC 20005, USA; Department of Anthropology, University of North Carolina—Chapel Hill, CB#3115, 301 Alumni Hall, 207 E. Cameron Avenue, Chapel Hill, NC 27599, USA; Department of Anthropology, Washington University in St. Louis, 1 Brookings Drive, Campus Box 1114, St. Louis, MO 63130, USA; Department of Anthropology, Washington University in St. Louis, 1 Brookings Drive, Campus Box 1114, St. Louis, MO 63130, USA

**Keywords:** evolutionary life history, child malnutrition, subsistence transition theory, dietary diversity, child dietary patterns, complementary feeding

## Abstract

Dietary patterns spanning millennia could inform contemporary public health nutrition. Children are largely absent from evidence describing diets throughout human evolution, despite prevalent malnutrition today signaling a potential genome-environment divergence. This systematic review aimed to identify dietary patterns of children ages 6 months to 10 years consumed before the widespread adoption of agriculture. Metrics of mention frequency (counts of food types reported) and food groups (globally standardized categories) were applied to: compare diets across subsistence modes [gatherer–hunter–fisher (GHF), early agriculture (EA) groups]; examine diet quality and diversity; and characterize differences by life course phase and environmental context defined using Köppen–Geiger climate zones. The review yielded child diet information from 95 cultural groups (52 from GHF; 43 from EA/mixed subsistence groups). Animal foods (terrestrial and aquatic) were the most frequently mentioned food groups in dietary patterns across subsistence modes, though at higher frequencies in GHF than in EA. A broad range of fruits, vegetables, roots and tubers were more common in GHF, while children from EA groups consumed more cereals than GHF, associated with poor health consequences as reported in some studies. Forty-eight studies compared diets across life course phases: 28 showed differences and 20 demonstrated similarities in child versus adult diets. Climate zone was a driver of food patterns provisioned from local ecosystems. Evidence from *Homo sapiens* evolution points to the need for nutrient-dense foods with high quality proteins and greater variety within and across food groups. Public health solutions could integrate these findings into food-based dietary guidelines for children.

## INTRODUCTION

Childhood requires optimal nutrition to sustain rapid growth and establish healthy metabolic processes that may endure a lifetime. The infant and juvenile phases of human life history differ from those of other primates in terms of phase duration, feeding patterns, brain growth, and social play dynamics [1]. Yet globally, many children are compromised nutritionally: 21.3% have stunted growth; 5.6% of children are overweight and millions are affected by nutrient deficiencies arising from poor quality diets [2]. Malnutrition in childhood can have dire consequences, including disease morbidity and mortality and compromised development—spanning cognitive, language, socio-emotional and motor developmental domains [3]. Suboptimal diets throughout the life course, but especially during childhood, contribute substantially to the global burden of disease. In 2017, poor quality diets led to 11 million deaths and 255 million disability-adjusted life years globally [4]. This systematic review was undertaken based on the premise that child dietary patterns today, dating back to increased reliance on agriculture, diverge from those of our evolutionary past with serious implications for health and development. Here, we aimed to synthesize evidence for child diets in *Homo sapiens* evolution with a view toward building from observational data to experimentally examine particular foods and patterns that could inform dietary guidelines and alleviate malnutrition in children today.

Dietary recommendations of macro- and micronutrients are based on evidence for maintaining health across the life course [5]. In recent years, there has been an effort to translate nutrient recommendations into culturally relevant food-based dietary guidelines, in recognition that nutrients interact with each other in the diet matrix and are metabolized differentially based on dietary patterns. These guidelines also recognize that people consume foods, not nutrients in isolation and need guidance on how to achieve a healthy and diverse diet. Further, there are substantial, yet-to-be-discovered compounds beyond the 150 nutrients and biochemical factors known to contribute to human health [6]. There is widespread recognition that diverse food systems have moved toward homogenized ones, with a mere three basic cereals (rice, maize and wheat) providing over half of calories consumed globally (60%). Similarly, the dietary contribution of terrestrial animal foods increasingly comes from a dwindling number of livestock breeds [7, 8]. Children from low-resource settings, in particular, consume high percentages of daily energy intakes from carbohydrates, increasing their risk of stunting and micronutrient deficiencies, while dietary patterns from the ‘nutrition transition’ occurring worldwide are characterized by increased fat, refined sugar and highly processed food intakes coinciding with increased chronic disease [9]. These rapid shifts in dietary patterns over the last century might also jeopardize children’s growth and development when in disagreement with a human physiology adapted to more ancient diets, as proposed by the genome-nutrition divergence framework [10].

Following Eaton and Konner’s work in 1985 [11], numerous attempts have been made to reconstruct ancestral diets of *Homo sapiens*, with an imagined singular ‘paleodiet’ dominating popular perspective. Others have recognized the complexity of dietary patterns with a deeper appreciation for the temporal and regional variations from *Homo erectus* to later members of the genus *Homo* [12, 13]. Despite the debate around the content of the ‘paleodiet’, multiple lines of evidence show the greatest dietary shifts followed widespread adoption of agriculture during the Neolithic era and later industrialization [11]. Skeletal evidence suggests that for many populations, agriculture was associated with a reduction in stature and lifespan, and an increase in infectious disease burden, as indexed by increasing frequency of skeletal markers associated with nutritional deficiencies, including porotic hyperostosis [14]. Evidence from changes in our genome does suggest the potential for plasticity and adaptations, yet precision around the timeframe remains unclear and may still take millennia [15].

In reconstructions of ancestral diets and health, children are largely absent from the literature [16]. Where present, the focus has been on breastfeeding and age of weaning among hominins with only minimal coverage of complementary feeding [Glossary Term 1] (Box 1) [17]. One review suggested that child complementary feeding diets pre-dating the agricultural revolution were more nutrient-dense relative to contemporary complementary feeding patterns, providing higher amounts of bioavailable zinc and iron [18]. A comprehensive review of ethnographies from ‘pre-industrial populations’ (defined by the authors as those ‘marginal to the emerging industrial capitalist world system at the time of ethnographic or ethnohistorical description’) examined breastfeeding practices and described ‘weaning’ foods [Glossary Term 2] (Box 1) [19]. We build on this work to more directly identify complementary feeding and childhood diets, appreciating the diversity that is central the evolution of *Homo sapiens*. Our systematic review aimed to identify foods from the diets of children ages 6 months to 10 years likely consumed prior to the widespread adoption of agriculture. Specific objectives were to: (i) characterize and contrast child dietary patterns across subsistence modes, specifically focusing on gatherer–hunter–fisher (GHF) compared to early agriculture (EA) societies; and (ii) conduct sub-group analyses to examine child dietary patterns based on: diet quality [ASF vs plant source foods (PSF)]; life course phase (child diet vs adult diets, complementary feeding vs post-weaning child); and environmental context (climate, geography).

Box 1. Glossary of terms

**Table:** 

*Child feeding terms*	
1. Complementary feeding period	The period when other solid foods or liquids are given to the child with continued breastfeeding.
2. Weaning foods	Foods consumed by infants in the transition to breastfeeding cessation.
*Pre-historic time periods covered in the review*	
3. Mesolithic era	Approximately 20 000–5000 years before present, depending on region.
4. Neolithic era	Approximately 10 000–3000 years before present, depending on region.
*Definitions of life course phases covered in the review, by discipline*	
5. Public Health	Public health institutions define early phases of the life course as: *infancy* (birth to 1 year); *toddler* (1–3 years); *childhood (1*–*10 years)* and *adolescence* (10–18 years) [98, 99].
6. Anthropology	Anthropologists use similar criteria to those used in public health to define life history periods demarcated by key growth and developmental milestones: *infancy* (birth to 36 months); *childhood* (3–6.9 years) and *juvenile* (7–10 girls, 7–12 boys) [1, 100].

## METHODS

Reporting for this review follows the guidelines of the Preferred Reporting Items for Systematic Reviews and Meta-Analysis (PRISMA) statement [20]. The review protocol was registered at the University of York’s International Prospective Register of Systematic Reviews (PROSPERO) and was published on 4/28/20 under the registration number CRD42020150123.

### Eligibility criteria

We included peer-reviewed papers and grey literature such as book chapters and dissertations describing child diets for subsistence populations who foraged wild foods through gathering, hunting, and fishing or EA [21]. We use the terminology that places ‘gatherer’ first in the phrase ‘gatherer–hunter–fisher’ to elevate this traditionally female role and to recognize the substantial contribution of gathering to dietary intakes [22]. EA was defined as mixed subsistence GHF and agriculture societies in the Mesolithic or Neolithic Ages [Glossary Terms 3 and 4] (Box 1) as well as those from contemporary ‘pre-industrial’ groups. Our criteria for child age range combined definitions from public health nutrition and anthropological studies of growth and development, including children ages 6 months to 10 years [Glossary Terms 5 and 6] (Box 1). We included studies with a range of designs and methods: stable isotope analyses of bone, teeth and hair; non-specific stress markers identified on skeletal remains; and ethnographies (Box 2). Ethnographies from modern-day foraging communities were included using a similar approach applied in key papers comparing the diets of Pleistocene versus Holocene hominin adults [11, 23–25]. GHF groups today differ from than those in the past in ways that may influence findings—the few remaining GHF populations today are often marginalized with restricted access to land and resources, subject to state-sanctioned violence and displacement, and generally use modern technologies (e.g. weapons) for food acquisition [26]. In some studies, archeological records of food types (e.g. middens of shells, botanical material or animal bones) may have supplemented the primary methods applied to characterize child diets but were not always described and, thus, not quantified here. There were no restrictions based on publication date or geographic location of the study. Articles were also limited to those written in English, French and Spanish. Exclusion criteria were for studies: without child diet information; describing for hominid species predating *Homo erectus*; and on contemporary, ‘pre-industrial’, subsistence groups exclusively dependent on agriculture.

### Search strategy

Articles included in this review were obtained from electronic databases outlined in Supplementary Table 1. Search terms included various combinations, truncations and synonyms of keywords: ‘infants’, ‘children’, ‘hunter gatherer’, ‘prehistoric’, and ‘diet’. Supplementary Table 1 details the full electronic search strategy.

### Study selection

Records were first merged across the database searches. The citations were subsequently exported and uploaded to Rayyan QCRI, an online systematic review application [27]. Prior to title and abstract screening, all duplicates were removed, resulting in a set of articles for the first round of screening in Rayyan QCRI. Two reviewers independently assessed all the citations for inclusion based on their titles and abstracts through a blinded process. Discrepancies from the first round of screening were resolved by a third reviewer, yielding articles considered eligible for a full-text review. The full-text review was conducted by two reviewers to further assess eligibility for inclusion, and discrepancies were resolved through team consensus. Through snowball sampling, we then identified additional literature applying the same eligibility criteria yielding peer-reviewed articles, books and other grey literature.

There was extensive heterogeneity across studies in terms of design, methods, sample sizes and child age, thereby limiting risk of bias assessments. Study designs, largely observational, were not amenable to the application of all GRADE criteria [28]. However, we invoked relevant GRADE domains to evaluate the quality of the included studies and to identify individual studies to highlight: (i) risk of bias; (ii) inconsistency and (iii) indirectness [28]. We examined different forms of bias (selection, reporting, survival, publication, etc.) present in study design. Moreover, we explored consistency of findings related to review objectives. Indirectness was examined first by assessing the extent to which studies met eligibility criteria and second, how relevant the studies were to review objectives.

Box 2. How study design and methods indicate child dietary intakes

**Table:** 

Carbon stable isotope ratios from bone and dentin collagen	δ^13^C values (i.e. the proportion of ^13^C in relation to the more abundant ^12^C isotope) are used to distinguish consumption levels of C_3_ versus C_4_ plants. C_3_ and C_4_ plants exhibit distinct carbon isotopic compositions, with C_3_ plants containing less ^13^C than C_4_ plants, enabling the reconstruction of dietary practices and the spread of C_4_ agricultural plants (e.g. millet and maize).
Nitrogen stable isotopes from bone and dentin collagen	δ^15^N values are used to characterize diets by trophic level [101]. The ratio between ^14^N and ^15^N varies predictably across trophic levels, such that samples from organisms at high trophic levels (e.g. carnivores) contain more ^15^N relative to ^14^N [102]. δ^15^N values indicate breastfeeding and weaning age, because a nursing infant is at a higher trophic level than its mother, and its nitrogen isotope values decrease during when complementary foods are introduced and during weaning [103]. Shifts in nitrogen isotopes have also historically been used to identify major dietary shifts, such as the transition from a human milk diet to one incorporating increasing amounts of the child/adult diet [104–106]. δ^15^N values may also represent severe malnutrition, or other forms of physiological stress and disease [107].
Analyses of teeth and protective enamel	Teeth are used to assess physiological stress (through analyses of enamel hypoplasia), losses in dietary quality (dental caries), and changes in diet consistency and food preparation [49]. Teeth are also used for stable isotope analysis where oxygen isotopes derived from carefully sampling teeth can be used to detect dietary shifts associated with weaning [108].
Ethnographies	Ethnographies from contemporary GHF groups are used to represent the dietary patterns of past and present foraging societies. Dietary data from ethnographies are captured with an array of methods including 24-hour dietary recalls in which study participants report detailed information on all meals consumed during the previous day, along with ingredients and methods that were used in meal preparations. Additionally, ethnographers may employ other observational methods which include following children on their foraging trips (also known as ‘focal person follows’); systematic scanning, counting, and weighing of prey or food samples brought to the camp from foraging and hunting trips; and structured behavioral observations of child feeding (also called focal child sampling).

### Data extraction and management

Data were extracted and are provided in Supplementary Table 2. For child diet, we first extracted information by compiling lists of foods mentioned as part of the child diet for sampled subsistence groups. Subsequently, we computed frequencies of these mentions across studies, adapting the method described by Sellen and Smay [19]. Foods were classified as part of the ‘complementary feeding diet’ when the children in the sample were known to also be breastfeeding. All other diets were classified as post-weaning or unknown breastfeeding status.

Child diets were next characterized by examining the frequencies of mentioned foods falling into the categories of ASF versus PSF. Standardized food groups were then used to categorize foods and broadly assess nutritional composition of the child diets: cereals; roots/tubers; vegetables; fruits; meat/poultry/offal; eggs; fish and seafood; pulses/legumes/nuts; milk/milk products; oils/fats; sugar/honey; and other [29]. Unspecified animal derivatives and insects were categorized in the meat/poultry/offal category, and all unspecified plants and plant parts were placed in the other category. We then applied FAO’s Guidelines for Measuring Household and Individual Dietary Diversity Household Dietary Diversity Score (HDDS) to compare overall dietary diversity across subsistence modes and environmental contexts [30]. Wilcoxon rank sum and Kruskal–Wallis tests were applied to compare HDDS values by different strata. Finally, we standardized mention frequencies by calculating percentages using the number of food group mentions out of total mentions across food groups in each sub-category—GHF, EA and climate zones.

Biogeographic information was extracted from studies and subsequently used to categorize the study site environment in two ways: (i) World Bank region [31] and (ii) Köppen–Geiger (KG) climate classification [32]. The World Bank region classifications are based on current geographic labels. In recognition that diets and food access in the evolutionary past of the genus *Homo* were environment-dependent, we also explored options for differentiating diets by biomes or ecosystems. We selected the KG climate classification as a widely applied system incorporating precipitation and temperature variables through time to define five main zones: (A) tropical (equatorial); (B) arid (dry); (C) temperate (warm/mild); (D) continental (snow) and (E) polar (alpine) [32, 33]. For each study, climate zones were assigned considering historical data relative to a certain area and to the time period most proximal to that referenced in the study. Thus, all studies based on contemporary ‘pre-industrial’ populations were analyzed according to the specific KG climate classification for 1976–2000 [32]. The remaining studies from the Neolithic and Mesolithic eras were analyzed according to the KG climate classification for the Mid-Holocene period from 5000 to 7000 years ago [33], recognizing that some samples fall outside this period when climate data were not available.

**Figure 1.**
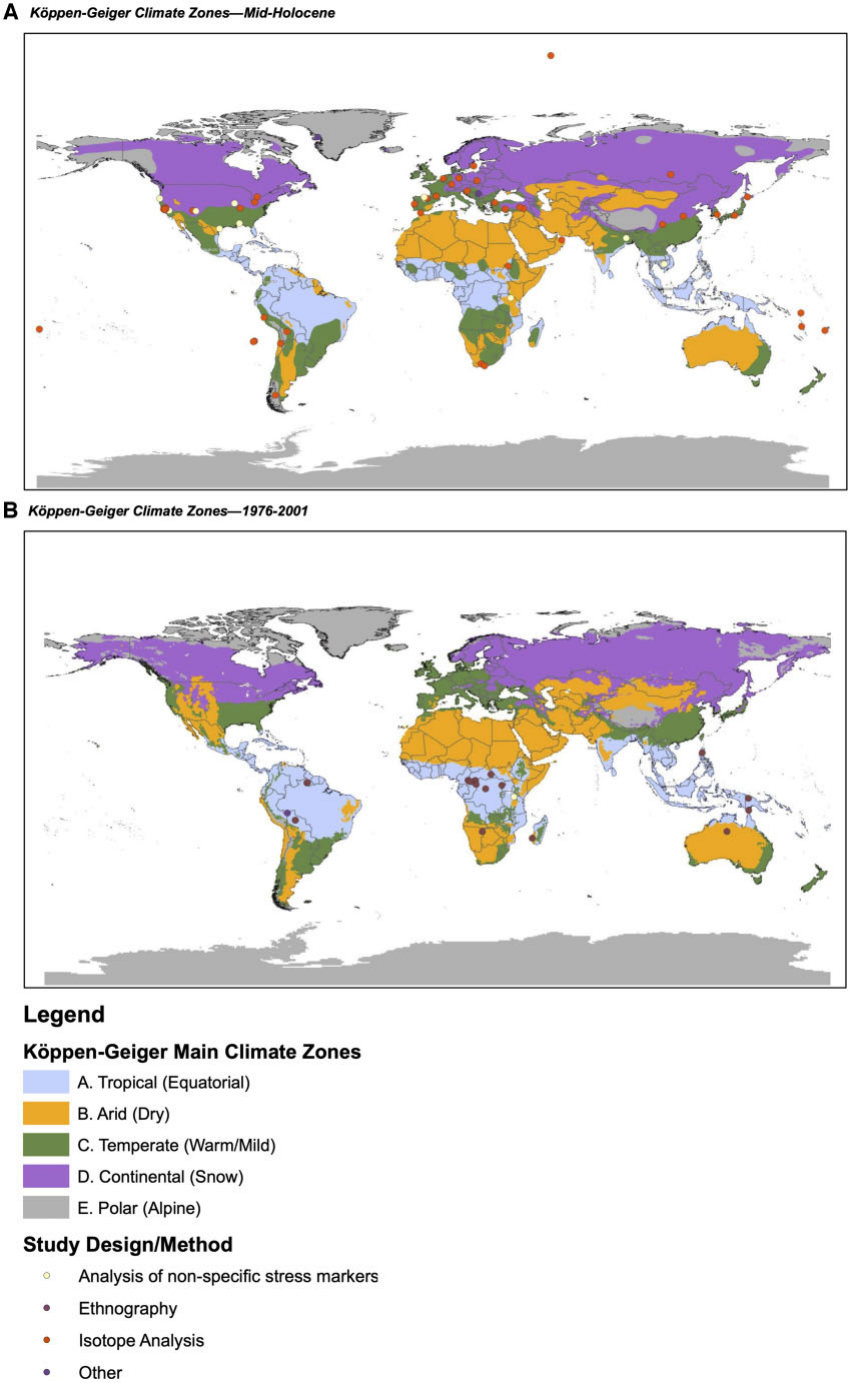
Geographic distribution of subsistence groups by Köppen–Geiger climate zones and study design/method. Map A represents subsistence groups from Neolithic and Mesolithic eras mapped according to Köppen–Geiger climate classification for the Mid-Holocene period from 5000 to 7000 years ago [33]. Map B represents contemporary ‘pre-industrial’ populations mapped according to Köppen–Geiger climate classifications for 1976–2000 [32]

To understand the geographic representation of the included studies, each was mapped using symbols representing study design (Fig. 1). We used ArcGIS [34] to geocode each site included in this review using GPS coordinates obtained from Google maps.

## RESULTS

There were 3435 records identified and screened; 269 full-text articles remained that met eligibility criteria (Supplementary Fig. 1). Authors (E.A.G., M.R., M.K.C. and L.L.I.) identified and screened an additional 17 relevant studies from references cited in the initial round of 76 articles, resulting in a total of 93 for the review.

The design and methods applied for the included studies are enumerated in Table 1, and sub-group characteristics in Table 2. Across the 93 studies, 95 distinct cultural groups were included in our analyses. Isotope studies comprised over half of the articles included (54.8%), followed by ethnographies (28.0%) and analyses of non-specific stress markers (14.0%). Approximately one-half (54.7%) of the groups practiced gathering, hunting and/or fishing. Fishing was observed in 37.9% of groups, while gathering and hunting activities were most prevalent at 78.9%. In terms of regional representation, sampled groups were mainly concentrated in Europe and Central Asia cumulatively (25.3%) and the Sub-Saharan African region (21.1%). South Asia and the Middle East and North Africa were underrepresented, with each region providing evidence on one (1.1%) cultural group.

**Table 1.** Research design and methods classification of included studies

Study design and methods	Complementary feeding diets^a^*N* (%)	All subsistence groups^b^*N* (%)
Isotope analysis	25 (54.3)	51 (54.8)
Ethnography	11 (23.9)	26 (28.0)
Non-specific stress marker analysis	8 (17.4)	13 (14.0)
Other^c^	2 (4.3)	3 (3.2)
Total	46 (100.0)	93 (100.0)

a Frequency (%) of complementary feeding diets reported in different sub-strata. Dietary patterns described in the subsistence group explicitly mentions ongoing breastfeeding with foods described.

b Frequency (%) of diets reported in different sub-strata among all subsistence groups (*n* = 95) mentioned in the full set of studies included in the review (*n* = 93). These values include those from the previous column of complementary feeding diets.

c The Other category included one genetic analysis (counted in Complementary feeding diets and all subsistence groups columns), one pharmacognostic analyses of fecal matter (counted in complementary feeding diets and all subsistence groups columns) and one analysis of bite marks on bone spoons (all subsistence groups column).

**Table 2.** Characteristics of included groups

Category	Sub-categories	Complementary feeding diets^a^*N* (%)	All subsistence groups^b^*N* (%)
Subsistence mode			
	Gatherer–hunter	14 (29.2)	32 (33.7)
	Gatherer–hunter–fisher	12 (25.0)	20 (21.1)
	Gatherer–hunter–fisher–agriculture	8 (16.7)	16 (16.8)
	Gatherer–hunter–agriculture	5 (10.4)	7 (7.4)
	Agriculture	9 (18.8)	20 (21.1)
Köppen–Geiger climate zones			
	A. Tropical (equatorial)	14 (29.2)	30 (31.6)
	B. Arid (dry)	6 (12.5)	12 (12.6)
	C. Temperate (warm/mild)	15 (31.3)	31 (32.6)
	D. Continental (snow)	10 (20.8)	19 (20.0)
	E. Polar (alpine)	3 (6.3)	3 (3.16)
World Bank regions			
	East Asia and Pacific	9 (18.8)	17 (17.9)
	Europe and Central Asia	6 (12.5)	24 (25.3)
	Latin America and Caribbean	13 (27.1)	16 (16.8)
	Middle East and North Africa	0 (0.0)	1 (1.1)
	North America	11 (22.9)	16 (16.8)
	South Asia	0 (0.0)	1 (1.1)
	Sub-Saharan Africa	9 (18.8)	20 (21.1)
Total		48 (100.0)	95 (100.0)

a Frequency (%) of complementary feeding diets reported in different sub-strata. Dietary patterns described in the subsistence group explicitly mentions ongoing breastfeeding with foods described.

b Frequency (%) of diets reported in different sub-strata among all subsistence groups (*n* = 95) mentioned in the full set of studies included in the review (*n* = 93). These values include those from the previous column of complementary feeding diets.

**Figure 2.**
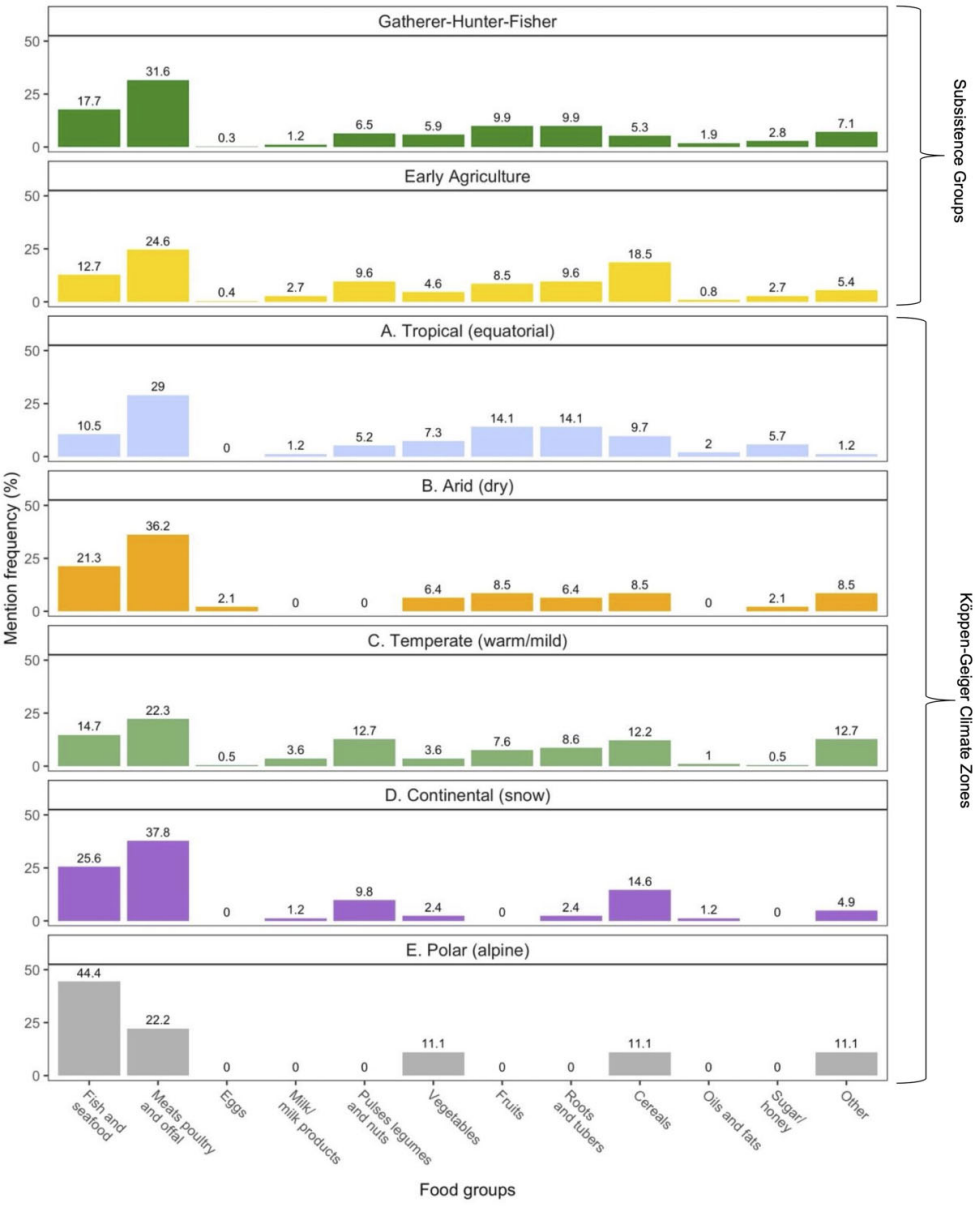
Food group frequencies (%), by subsistence mode and Köppen–Geiger climate zone. Percentages were calculated as number of mentions over total number of mentions for the specific subsistence mode or climate zone. Foods were assigned to food groups using FAO’s Guidelines for Measuring Household and Individual Dietary Diversity [29, 30]

*Subsistence mode*. In general, we found higher mention frequencies for animal foods containing high-quality proteins in children coming from GHF societies compared to EA. The most commonly mentioned food group in the child diets was the meat, poultry, offal category [*n* = 102 (31.6%) for GHF, *n* = 64 (24.6%) for EA] (Table 3; Fig. 2). Insects appeared 11 times for GHF compared to only once for EA. Fish and seafood followed in terms of proportional frequency of mentions for GHF [*n* = 57 (17.7%), though for EA groups, cereals were the second most frequent food mentioned [*n* = 48 (18.5%)]. We observed a variety of foods reported in the fish and seafood group, with aquatic mammals appearing more frequently for GHF than EA. Milk and eggs were less common than other ASF. Honey, as an animal product, appeared more often among GHF children than EA.

**Table 3.** Child evolutionary diets, by subsistence mode

Food groups	Gatherer–hunter–fisher^a^	Early agriculture^b^
*Fish and seafood*	Aquatic Mammals (12)	Aquatic Mammals (2)
	Finfish (31)	Finfish (18)
	Mollusks and Crustaceans (9)	Mollusks and Crustaceans (7)
	Unspecified fish/seafood (4)	Unspecified fish/seafood (6)
	Roe (1)	
	***N* = 57 (17.7%)**	***N* = 33 (12.7%)**
*Meats, poultry, offal*	Birds (10)	Birds (5)
	Insects (11)	Insects (1)
	Mammals (47)	Mammals (37)
	Rodents (9)	Bats (2)
	Ungulates (29)	Rodents (2)
	Other Mammals (9)	Ungulates (27)
		Other mammals (6)
	Reptiles (11)	Reptiles (4)
	Unspecified animals (8)	Unspecified animals (5)
	Meat (animal source unspecified) (12)	Meat (animal source unspecified) (12)
	Amphibians (1)	
	Offal/Animal derivatives (2)	
	***N* = 102 (31.6%)**	***N* = 64 (24.6%)**
*Eggs* (avian)^c^	Bird eggs (1)	Bird eggs (1)
	***N* = 1 (0.3%)**	***N* = 1 (0.4%)**
*Milk and milk products*	Milk (4)	Milk (3)
		Unspecified dairy products (4)
	***N* = 4 (1.2%)**	***N* = 7 (2.7%)**
*Pulses, legumes, nuts*	Nuts (9)	Nuts (14)
	Acorns (4)	Acorns (2)
	Unspecified nuts (5)	Almonds (1)
		Hazelnuts (1)
		Peanuts (1)
	Seeds (11)	Pistachios (2)
	Beans (3)	Unspecified nuts (7)
	Peas (1)	
	Grass and other plant seeds (5)	Seeds (10)
	Unspecified seeds (2)	Beans (3)
		Peas (3)
	Unspecified legume (1)	Lentils (3)
		Unspecified seeds (1)
		Unspecified pulse (1)
	***N* = 21 (6.5%)**	***N* = 25 (9.6%)**
*Vegetables*	Allium/onions (1)	Leafy greens (2)
	Leafy greens (7)	Marine plants (1)
	Marine plants (2)	Mushrooms (2)
	Mushrooms (2)	Cactus (1)
	Squash (2)	Unspecified vegetables (4)
	Unspecified vegetables (5)	Hot pepper sauce (2)
	***N* = 19 (5.9%)**	***N* = 12 (4.6%)**
*Fruits*	Baobab fruit (4)	Apples (1)
	Berries (5)	Berries (2)
	Breadfruit (1)	Breadfruit (1)
	Citrus (1)	Coconut (1)
	Coconut (1)	Figs (1)
	Mango (1)	Opi fruit (1)
	Pandanus fruit (1)	Pears (1)
	Plantains/bananas (4)	Plantains/bananas (8)
	Melon (2)	Unspecified fruits (6)
	Tamarind (1)	
	Unspecified fruits (11)	
	***N* = 32 (9.9%)**	***N* = 22 (8.5%)**
*Roots and tubers*	Cassava/Manioc (6)	Cassava/Manioc (7)
	Unspecified root/tuber (11)	Kava (1)
	Sago (2)	Sweet potato (2)
	Sweet potato (3)	Taro (6)
	Taro (5)	Yams (6)
	Yams (5)	Unspecified root/tuber (3)
	***N* = 32 (9.9%)**	***N* = 25 (9.6%)**
*Cereals*	Maize/corn (6)	Barley (4)
	Rice (5)	Kiwicha (1)
	Sorghum (1)	Maize/Corn (14)
	Other grasses (3)	Millet (4)
	Unspecified cereal (2)	Processed foods (3)
		Rice (9)
		Wheat (6)
		Unspecified cereal (7)
	***N* = 17 (5.3%)**	***N* = 48 (18.5%)**
*Oils and fats*	Animal fats (2)	Vegetable/cooking oil (1)
	Palm nuts/palm oil (1)	Palm nuts/palm oil (1)
	Vegetable/cooking oil (2)	
	Unspecified oil/fat (1)	
	***N* = 6 (1.9%)**	***N* = 2 (0.8%)**
*Sugar/honey*	Sugar/sugarcane (2)	Sugar/sugarcane (4)
	Honey (7)	Honey (3)
	***N* = 9 (2.8%)**	***N* = 7 (2.7%)**
*Other*	C3 plants (5)	Aquatic resources (1)
	Herbal teas (2)	C3 plants (4)
	Miscellaneous plants (12)	C4 plants (4)
	Unspecified foods (5)	Salt (1)
		Herbal teas (1)
		Miscellaneous plants (2)
		Unspecified foods (2)
	***N* = 23 (7.1%)**	***N* = 14 (5.4%)**

a The **gatherer**–**hunter**–**fisher** group is comprised of the *gatherer*–*hunter* and *gatherer*–*hunter*–*fisher* sub-categories. *N* = frequency of mentions in food group category for GHF groups (% of total mentions for GHF).

b The **agriculture** group is comprised of the *gatherer*–*hunter*–*agriculture*, *gatherer*–*hunter*–*fisher*–*agriculture* and *agriculture* sub-categories. *N* = frequency of mentions in food group category for EA groups (% of total mentions for EA).

c The food group is titled *Eggs*, however we specify avian eggs since egg types appear elsewhere in other food groups (e.g. roe).

Bolded values represent N, total number of mentions for each food group according to subsistence mode.

For plant foods, we again observed notable differences by subsistence mode. Roots and tubers were the predominant carbohydrate-rich plant food for GHF [*n* = 32 (9.9%) while cereals (maize/corn, rice, wheat etc.) emerged more often in EA [*n* = 48 (18.5%)]. Pulses, legumes, and nuts containing both protein and carbohydrates were also observed in marginally higher frequencies for EA [*n* = 25 (9.6%)] compared to GHF [*n* = 21 (6.5%)]. Both GHF and EA groups showed common nut consumption, with a more diverse array of types observed among the EA. Fruits and vegetables, foods with multiple micronutrients, were again observed at higher mention frequency in GHF compared to EA. There were non-significant differences in HDDS by subsistence mode (*P* = 0.89 by Wilcoxon rank sum test), and the median HDDS for both groups was 3 (IQR = 3).

*Life course phase*. Of the 95 cultural groups examined in the review, 48 (50.5%) had information on complementary feeding diets (Table 2; Supplementary Tables 4 and 5). Of the articles comparing child diets with adult diets, 21 reported differences and 20 showed similar diets in children and adults in a single time-frame (inter-individual analyses). The remaining seven studies examined longitudinal dietary changes over the life course in individuals, demonstrating variations in diet during certain phases of the life course (intra-individual analyses). Notable differences in the complementary feeding diets emerged for some food groups, by subsistence mode. While there was higher fish and seafood frequencies for GHF complementary feeding diets (19.6% vs 17.7%) compared to the larger pool of studies, there was lower mention frequency for the meat, poultry and offal (21.6% vs 31.6%). Mention of cereals for EA groups was higher in complementary feeding diet compared to the larger pool (27.3% vs 18.5%). Three studies showed that child foraging behaviors for easily obtained foods resulted in dietary patterns at lower trophic levels (e.g. small vertebrates, shellfish, and plants) compared to those for adults [35–37]. In other studies, adults were reported to consume increased or higher amounts of ASF in absolute terms compared to children [38–40].

Although not explicitly stated in the original review objectives, we briefly describe results regarding differences by sex of the child. Comparisons of dietary patterns by sex of the child were reported in 14 studies: six showed differences in consumption patterns of girls compared to boys; and eight found no differences. Gendered foraging behaviors were suggested to have explained sex-based dietary patterns, with males consuming foods at higher trophic levels (more ASF) from hunting and females at lower trophic levels (plants) from gathering or cultivation [41–43].

*Environmental context*. Overall, the studies included in this review were concentrated in temperate (warm/mild), tropical (equatorial) zones and continental (snow) zones (Table 2). Studies analyzing child diets using isotopes and non-specific stress markers from Paleolithic and Neolithic periods were positioned primarily in temperate and continental climates, while the ethnographies came largely from Sub-Saharan Africa and East Asia and Pacific regions in tropical and sometimes arid zones today (Fig. 1). All climate zones showed high mention frequencies for ASF relative to other food groups (Fig. 2; Table 4). There were statistically significant differences in HDDS by climate zone (*P* < 0.001 by Kruskal–Wallis test). Median HDDS scores were: tropical (equatorial), 4 (IQR = 3); temperate (warm/mild), 3 (IQR = 3); polar (alpine), 4 (IQR = 0.5); arid (dry), 3 (IQR = 1.5) and continental (snow), 3 (IQR = 1.5). Given differences in design and methods, however, results from these statistics should be interpreted with caution.

**Table 4.** Child evolutionary diet, by Köppen–Geiger climate zones

	Tropical (equatorial)^a^	Arid (dry)^b^	Temperate (mild/warm)^c^	Continental (snow)^d^	Polar (alpine)^e^
*Fish and seafood*	Finfish (17)	Aquatic Mammals (3)	Aquatic Mammals (4)	Aquatic Mammals (5)	Aquatic Mammals (2)
	Mollusks and Crustaceans (9)	Finfish (4)	Finfish (15)	Finfish (11)	Finfish (2)
		Mollusks and Crustaceans (3)	Mollusks and Crustaceans (3)	Unspecified seafood (4)	
			Unspecified fish/ seafood (7)	Roe (1)	
	***N* = 26 (10.5%)**	***N* = 10 (21.3%)**	***N* = 29 (14.7%)**	***N* = 21 (25.6%)**	***N* = 4 (44.4%)**
*Meats, poultry, offal*	Birds (5)	Birds (4)	Amphibians (1)	Birds (2)	Mammals (1)
	Insects (10)	Insects (1)	Birds (4)	Meat (animal source unspecified) (3)	Ungulates (1)
	Mammals (36)	Mammals (3)	Insects (1)		Meat (animal source unspecified) (1)
	Bats (2) Rodents (9) Ungulates (18) Other Mammals (7)	Ungulates (2) Other Mammals (1)	Mammals (21) Rodents (2) Ungulates (16) Other Mammals (3)	Mammals (23) Ungulates (19) Other Mammals (4)	
	Meat (animal source unspecified) (11)	Meat (animal source unspecified) (3)	Meat (animal source unspecified) (6)	Unspecified Animals (3)	
	Reptiles (3)	Reptiles (6)	Offal/Animal derivatives (2)		
	Unspecified Animals (7)		Reptiles (6)		
			Unspecified Animals (3)		
	***N* = 72 (29.0%)**	***N* = 17 (36.2%)**	***N* = 44 (22.3%)**	***N* = 31 (37.8%)**	***N* = 2 (22.2%)**
*Eggs (avian)*		Bird eggs (1)	Bird eggs (1)		
	***N* = 0 (0.0%)**	***N* = 1 (2.1%)**	***N* = 1 (0.5%)**	***N* = 0 (0.0%)**	***N* = 0 (0.0%)**
*Milk and milk product*	Milk (2)		Milk (4)	Milk (1)	
	Unspecified dairy products (1)		Unspecified dairy products (3)		
	***N* = 3 (1.2%)**	***N* = 0 (0.0%)**	***N* = 7 (3.6%)**	***N* = 1 (1.2%)**	***N* = 0 (0.0%)**
*Pulses, legumes, nuts*	Nuts (11)		Nuts (10)	Nuts (2)	
	Peanuts (1) Unspecified nuts (10)	***N* = 0 (0.0%)**	Acorns (6) Almonds (1) Hazelnuts (1) Pistachios (1) Unspecified nuts (1)	Pistachios (1) Unspecified nuts (1)	
	Seeds (1) Beans (1)		Seeds (13) Beans (2) Lentils (2) Peas (2) Grass and other plant seeds (5) Unspecified seeds (2)	Seeds (6) Beans (3) Lentils (1) Peas (2)	
			Unspecified legume (1)		
			Unspecified pulse (1)		
	***N* = 13 (5.2%)**		***N* = 25 (12.7%)**	***N* = 8 (9.8%)**	***N* = 0 (0.0%)**
*Vegetables*	Leafy Greens (6)	Allium/Onion (1)	Cactus (1)	Marine plants (1)	Leafy greens (1)
	Mushrooms (4)	Squash (1)	Leafy greens (2)	Unspecified vegetables (1)	
	Hot pepper sauce (2)	Unspecified vegetables (1)	Marine plants (2)		
	Unspecified vegetables (6)		Squash (1)		
			Unspecified vegetables (1)		
	***N* = 18 (7.3%)**	***N* = 3 (6.4%)**	***N* = 7 (3.6%)**	***N* = 2 (2.4%)**	***N* = 1 (11.1%)**
*Fruits*	Baobab fruit (4)	Berries (2)	Apples (1)		
	Berries (2)	Melon (1)	Berries (3)		
	Breadfruit (1)	Unspecified fruits (1)	Breadfruit (1)		
	Citrus (1)		Coconut (1)		
	Coconut (1)		Figs (1)		
	Mango (1)		Pears (1)		
	Melon (1)		Unspecified Fruits (7)		
	Opi fruit (1)				
	Pandanus fruit (1)				
	Plantains/Banana (12)				
	Tamarind (1)				
	Unspecified fruits (9)				
	***N* = 35 (14.1%)**	***N* = 4 (8.5%)**	***N* = 15 (7.6%)**	***N* = 0 (0.0%)**	***N* = 0 (0.0%)**
*Roots and tubers*	Cassava/Manioc (11)	Yams (1)	Cassava/Manioc (1)	Cassava/Manioc (1)	
	Sago (2)	Unspecified root/tuber (2)	Kava (1)	Unspecified root/tuber (1)	
	Sweet potato (1)		Sweet potato (1)		
	Taro (4)		Taro (7)		
	Yams (9)		Yams (1)		
	Unspecified root/tuber (8)		Unspecified Root/Tuber (3)		
	***N* = 35 (14.1%)**	***N* = 3 (6.4%)**	***N* = 17 (8.6%)**	***N* = 2 (2.4%)**	***N* = 0 (0.0%)**
*Cereals*	Maize (7)	Maize (3)	Barley (3)	Barley (1)	Unspecified cereal (1)
	Processed foods (3)	Other grasses (1)	Kiwicha (1)	Maize (4)	
	Rice (12)		Maize (6)	Millet (2)	
	Sorghum (1)		Millet (2)	Rice (1)	
	Unspecified cereal (1)		Rice (1)	Wheat (2)	
			Wheat (4)	Unspecified cereal (2)	
			Other grasses (2)		
			Unspecified Cereal (5)		
	***N* = 24 (9.7%)**	***N* = 4 (8.5%)**	***N* = 24 (12.2%)**	***N* = 12 (14.6%)**	***N* = 1 (11.1%)**
*Oils and fats*	Palm nuts/palm oil (2)		Animal fats (2)	Unspecified fats (1)	
	Vegetable/cooking oil (3)				
	***N* = 5 (2.0%)**	***N* = 0 (0.0%)**	***N* = 2 (1.0%)**	***N* = 1 (1.2%)**	***N* = 0 (0.0%)**
*Honey and sugar*	Honey (10)	Sugar/sugar cane (1)	Sugar/sugar cane (1)		
	Sugar/sugar cane (4)				
	***N* = 14 (5.7%)**	***N* = 1 (2.1%)**	***N* = 1 (0.5%)**	***N* = 0 (0.0%)**	***N* = 0 (0.0%)**
*Other*	Miscellaneous plants (2)	C3 plants (1)	Aquatic resources (1)	C3 plants (3)	Miscellaneous plants (1)
	Salt (1)	C4 plants (2)	C3 plants (4)	C4 plants (1)	
		Unspecified foods (1)	C4 plants (1)		
			Herbal teas (3)		
			Miscellaneous plants (11)		
			Unspecified foods (5)		
	***N* = 3 (1.2%)**	***N* = 4 (8.5%)**	***N* = 25 (12.7%)**	***N* = 4 (4.9%)**	***N* = 1 (11.1%)**

a *N* = frequency of mentions in food group category for groups living in tropical (% of total mentions for tropical).

b *N* = frequency of mentions in food group category for groups living in arid (% of total mentions for arid).

c *N* = frequency of mentions in food group category for groups living in temperate (% of total mentions for temperate).

d *N* = frequency of mentions in food group category for groups living in continental (% of total mentions for continental).

e *N* = frequency of mentions in food group category for groups living in polar (% of total mentions for polar).

Bolded values represent N, total number of mentions for each food group according to Köppen–Geiger climate zones.

### Results of individual studies

We identified eight studies that most directly addressed the review objectives and yielded high-quality evidence with low risk of bias.

*Subsistence mode.* In a study of the Caribbean region, Chinique de Armas and Pestle [44] compared dietary patterns in children ranging in age from 0 to 10 years (*n* = 88) across three distinct subsistence modes: GHF; horticulturalists and agriculturalists. The authors suggested that the high values of nitrogen enrichment in the GHF group indicated that meat, marine and riverine foods were included in the infant or young child diets, possibly premasticated before the eruption of first teeth. The GHF individuals showed greater intra-population isotope value variability than was evident in the horticulture and agriculturalist groups, pointing to a wider range of food options.

Shuler *et al*.’s [45] study of the central Tombigee valley of present-day Alabama and Mississippi analyzed skeletal remains from 11 sites to characterize dietary patterns and health conditions associated with the transition to agriculture from 500 to 1200 AD. The Late Woodland populations using foraging and horticulture showed greater dietary diversity and better health outcomes compared to the farmstead (EA) populations, who relied heavily on maize. Remains of the farmstead children indicated nutritional deficiencies and infection, evident from the high prevalence of linear enamel hypoplasia (60.9%).

In one study by Ungar *et al*. [46], they explicitly investigated differences in weaning diets by subsistence mode among the Hadza of Tanzania. Authors compared prevalence of linear enamel hypoplasia, indicating metabolic disruptions and nutritional stress, in children with three dietary patterns: bush weaning diet; village weaning diet and a transition group. The bush diet included (percent total energy): meat (31–32%); berries, figs, drupes and legumes (18–22%); tubers (13–18%); baobab fruit (4–18%); honey and larvae (11–14%); and other agriculture products (1–8%). Meat type was highly varied including a large array of birds, greater than 200 species, and game mammals. By contrast, the village weaning diet consisted of rice, maize, beans and foods obtained in the markets such as onions, avocados, amaranth greens and domesticated meat, with a predominance of PSF. Linear enamel hypoplasia was significantly higher in the village group compared to the bush group.

*Life course phase*. Using dentin and bone isotopic analyses, Stantis *et al.* [37] presented evidence from the island of Viti Levu in Fiji on dietary differences between children and adults. The investigators found that child diets likely included more terrestrial plants and mangrove shellfish compared to those of adults, though, overall, the community relied heavily on marine foods, especially those from lower trophic levels.

Age differences in dietary patterns were directly examined by Bullington [47] in a study from the lower Illinois River Valley among 36 individuals ages 6–27 months. This study compared dental microwear in individuals living during the Middle Woodland period (50 BC to 250 AD) practicing extensive horticulture with GHF to those from the Mississippian period (1000–1350 AD) with agriculture primarily [47]. There were no differences by age in total feature frequency (scratch and pit frequency) and enamel characteristics for the Middle Woodland period, but increasing wear with age was evident in the Mississippian juveniles. The author suggested that child complementary feeding diets in the Middle Woodland period were more variable including harder foods earlier in life, whereas Mississippian juveniles primarily consumed maize, which was of a softer and more homogeneous consistency.

*Environmental context*. Evidence from island populations suggests that child diets were directly tied to local conditions. Kinaston *et al.*’s [48] study from Uripiv Island in Vanuatu applied stable isotopes to examine dietary changes with increasing intensification of horticultural and arboricultural systems beginning in the Lapita period (2600–2500 BP) through post-Lapita (2500–2000 BP) and finally, to late prehistoric/historic (300–150 BP). During the Lapita periods, there was broad spectrum foraging in both children and adults. With increasing horticultural practices, adult remains showed a move toward more terrestrial diets combined with marine resources from lower trophic levels. While remains from children were few, they showed higher concentrations of δ^15^N values compared to those of adults, likely arising from more marine and mangrove organisms in their diets.

Temple’s [49] study in Japan directly investigated differences in stress markers indicated by dental defects in association with geographic variation among foragers during the Jōmon period (5000 through 2300 BP). The author hypothesized that increasing population density and reliance on plant-based foods in western Japan would increase stress and secondarily lead to shorter stature compared to groups in eastern Japan where diets were based predominantly on marine harvesting and terrestrial mammal hunting systems. No differences were found in hypoplasia frequencies geographically, but there were decreases in stature from Middle Jōmon to Late Jōmon periods that were likely attributed to dietary stress.

In a study of skeletal remains in Eastern Siberia, Temple [50] highlighted the effects of nutritional stress from seasonality and food availability through analyses of femur length and body mass estimation. Comparing foraging populations in the Early (8000–6800 BP) to the Late (6000–5200 BP) Neolithic period, the study showed longer femur length and greater body mass in the latter when there was a wider dietary breadth of foods present—including freshwater fish, seal and terrestrial mammals. The Early Neolithic period in this region was cold and dry with high seasonal availability of foods. During both periods, femoral stunting and body mass wasting was evident at breastfeeding cessation and through adolescence.

## DISCUSSION

This systematic review aimed to identify foods consumed by children through ∼2 million years of hominin evolutionary history. Building from subsistence transition theory and the genome-nutrition divergence framework, we posited that dietary patterns would reveal differences among children in GHF compared to EA populations and that the seeming disconnect between dietary patterns and genomic expectations might help explain why child malnutrition is so prevalent today [10, 51, 52]. Our findings showed consistently higher frequency of the following food groups in GHF compared to EA: animal foods (both terrestrial and aquatic); roots and tubers; fruits; and vegetables. Among those studies examining life course differences, over half (58%) revealed child diets diverged from adults. Climate zone and local ecosystems drove the selection of foods in child dietary patterns.

As expected, there was no single prototype child diet. Instead, we observed a wide array of foods consumed during the infancy and childhood periods particularly in the GHF groups. Indeed, the median HDDS did not differ between GHF and EA groups. We applied HDDS as a standardized approach for assessing dietary diversity, however, it is important to acknowledge its limitations in capturing the full range of foods. Dietary diversity is widely recommended in dietary guidelines today, but these guidelines often lack specificity with respect to dietary patterns within food groups and rarely consider the implications of species richness and biodiversity for a sufficiently diverse diet [53]. In human evolution, a broader dietary spectrum is thought to have ensured survival particularly in the context of seasonality, food uncertainties and multiple environmental contexts around the world, though there is some debate for advantages conferred by being a specialist versus generalist [54]. Evidence suggests the solution may lie in the combination of the two, at least in modern foraging populations [55]. More research is needed to re-conceptualize dietary diversity for children today.

### Child dietary patterns and specific food types

In public health nutrition today, there has been a move toward developing food-based dietary guidelines for individuals and families, although countries still rely heavily on nutrient intake recommendations to guide policy [56]. This derives from evidence for nutrient interactions within the food matrix and the dynamic interplay of a multitude of bioactive factors in whole diets. Moreover, dietary guidelines consider the fact that people eat food, not nutrients, and therefore need guidance for making healthy food choices. Here, we highlight specific foods and food groups that emerged repeatedly in GHF dietary patterns compared to EA to point to potential nutrient composition differences, fully acknowledging the complexity of many unknown biochemical and environmental exposures.

Animal foods were present in the diets of nearly all GHF reviewed and showed reduced frequency in the EA groups in both absolute and proportional values. ASF, relative to PSF, contain high-quality proteins, as measured by the digestible indispensable amino acid score, and several critical limiting nutrients in highly bioavailable forms [57]. However, ASF are often absent from contemporary child diets in low-resource settings [58]. Randomized controlled trials testing ASF early in the complementary feeding period show their importance for child growth and development [59–61]. The continual presence of ASF in child diets was consistent with what has previously been shown in adult GHF diets, including the variability across ecosystems [24, 26]. In public health today, dietary guidelines in adults have encouraged reduced ASF intakes to reduce risk of chronic non-communicable diseases and minimize environmental impacts [62]. Again, child diets have largely been absent in this discourse despite unique nutrient requirements to support rapid growth and development. Indeed, some evidence suggests the potential for harms associated with vegan and vegetarian diets in young children [63–65].

Among the ASF foods appearing in child diets, a range of fish and other seafoods were frequently mentioned in the GHF diets, including mollusks and crustaceans easily foraged by children [66]. These foods provide critically important macronutrients such as docosahexaenoic acid and the micronutrients iron, zinc and iodine, which are vital for brain development. Recent evidence highlights the potential for aquatic foods in assuaging child malnutrition problems globally [67, 68]. Insects also commonly appeared in our review of the literature, perhaps in part due to the ease of foraging by children. These foods may also provide high quality proteins, lipids, minerals and other bioactive compounds shown to positively impact young children’s health [69–71]. Honey, an insect product, is an energy-dense food touted for its content of flavonoids and phenolic acids which can confer antioxidant, antibacterial and anti-inflammatory action in human health and may contain other child health-promoting compounds such as oligosaccharides [72]. Despite theories positing that eggs, particularly those of shore birds, were part of the evolutionary diet [73, 74] and recent evidence for positive impacts on child nutrition [60], our review found infrequent mention of eggs in child diets. This may, in part, be explained by the food’s fragility in the fossil record compared to evidence that remains for meat (e.g. cut-marks in animal bones) or seafood (e.g. shell middens) consumption. Milk and other dairy products also showed low mention frequency, unsurprisingly, given that animal domestication dates to ∼12 000 years ago [10].

Some differences in PSF groups—energy-dense roots/tubers and cereals; fruits; and vegetables—were also evident between GHF and EA. Cereals such as maize, rice, wheat, among others were reported at considerably higher frequency in the EA groups than GHF children. Children from GHF groups consumed an array of roots and tubers (yams, sweet potatoes, taro, sago, kava, cassava/manioc) and starchy fruits (plantains/bananas or baobab fruit) at greater absolute frequency than EA children, though the standardized percentages were similar in the two groups. These foods provide calorie-dense carbohydrates, carotenoids, vitamin C and phenolics [75].

Our review of individual studies showed that the cereal-predominant dietary pattern was associated with nutritional deficiencies and infection, and linear enamel hypoplasia [45, 46]. Today, cereals are more commonly the staple carbohydrate-dense food in diets compared to roots and tubers [76]. The cereal-based diet can be limiting in terms of many micronutrients, essential fatty acids and proteins, required for healthy growth in children [77]. Further, maize, in particular, contains phytates and other compounds that interfere with the absorption of minerals necessary for child growth [77, 78]. Thus, the increased dependence on cereal-based foods globally may partly explain compromised child growth patterns and interaction effects within the diet matrix [79]. Modern diets in children diverge from the EA diets in the past with the added exposures of ultra-processed carbohydrates and foods more generally, further heightening risks of malnutrition [80, 81].

Berries, already recognized for their antioxidant properties, emerged as frequent fruit sources in child diets. The sweet flavor and fructose sugar in berries also confers the benefit of calories and palatability for children. For both EA and GHF groups, a wider array of fruits—compared to a less diverse group of vegetable types—were mentioned. This potentially reflects investigators’ ability to more easily distinguish different fruit types than non-specific leafy greens. A variety of nuts and seeds were also described: acorns, almonds, hazelnuts, peanuts, pistachios and several other unspecified nuts. Nuts and seeds are less appreciated today, in part due to allergies prevalent in some regions of the world and concerns about texture for young children [82, 83]. Fungi, a distinct kingdom from plantae in taxonomic classification, appeared as mushrooms in child diets and could have been present in fermented foods. In addition to the foods that are actually mentioned, we wanted to highlight the near absence of other foods in contemporary GHF and EA groups, such as refined sugars and highly processed foods known to be associated with poor child health today [84].

### Life course patterns

We also aimed to explore dietary patterns by life course phase, specifically during infancy and childhood. In public health nutrition, the complementary feeding period defined as 6–24 months, falls within the ‘first 1000 days of life’ and is considered critical for establishing lifelong growth and developmental trajectories [85]. It is a highly vulnerable time for developing malnutrition as other foods beyond breast milk are introduced, and infants are increasingly exposed to infectious disease agents and environmental toxins. Today, dietary guidelines in many countries include ‘special nutrition considerations’ during childhood to accommodate rapid growth and these additional risks [86, 87]. We identified studies comparing adult and child cross-sectionally and others examining changes in diet longitudinally in the same individuals. Over half (58.3%) of these studies found differences in child versus adult, and the remaining (41.7%) showed child diets to be largely reflective of adult diets, consistent with a previous review [19]. While commonalities with adult diet would enable the extrapolation of evidence from adults in evolution, the nutritional demands of growth and development likely necessitates distinct guidelines for children today. This review’s focus on life course patterns demonstrated an inextricable link between child development and nutrition. For example, Stantis *et al.*’s [37] study from Fiji showed child diets likely included more terrestrial plants and mangrove shellfish compared to adults, potentially indicating the presence of child-specific foraging patterns. Child food preferences could be expressed and enabled by the foraging lifestyle, as suggested in Chinique de Armas and Pestle’s study from the Caribbean [44].

### Environmental context: ecosystem-derived foods

For most of our evolutionary past, *Homo sapiens* were embedded within ecosystems participating as one of many organisms in a dynamic food web. While this makes sense intuitively given limited technologies and mobility in the past and highlights plasticity in *Homo sapiens*, it points to a deeper connection between food and nature for children than what exists today. Today, increasingly homogenized and global food systems enable populations to disassociate from local environments and more easily access ultra-processed and evolutionarily novel food items. In some Indigenous communities today, traditional diets have been recognized for environmental sustainability, though unfortunately, substantial shifts away from these patterns are evident in these populations as well [88, 89]. Two studies in the review were from island communities and illustrative of ecosystem synchrony. Uripiv Island in Vanuatu is surrounded by a fringing reef and seagrass meadow, which harbors turtles, echinoderms, various shellfish and marine mammals [48]. Mangrove forests at the site also provide shelter for shellfish, crustaceans, bats and birds. Authors suggest these easily accessible foods might have enabled foraging of aquatic foods by children. Child diets from Stantis *et al.*’s [37] study in Viti Levu, Fiji were also found to be comprised of lower trophic foods such as shellfish from the marine ecosystem, mangrove forests and a lagoon surrounded by a fringing reef [37]. There have been recent movements to promote locally grown or raised diets, such as the Mediterranean or Nordic diets, for the dual purposes of health impacts and sustainability [90, 91]. There has also been increasing evidence for how horticulture or garden-based interventions can serve as a means to improving child nutrition and establishing healthy dietary patterns later in life [92, 93, 94]. Our review showed some positive effects arising from the practice of horticulture in combination with foraging, potentially adding more variation to the diet.

### Limitations and strengths

Heterogeneity in methods across studies presented challenges for comparative analyses, although this enabled a larger pool of studies and the opportunity to validate findings across multiple disciplines. Studies using hominin remains generally had small sample sizes, potentially precluding population representation. Mortality bias may have been present in the sub-adult remains due to premature child deaths, complicated by the ‘osteological paradox’ that children died early for reasons not evident in the skeleton [95]. Individuals studied could have perished from malnutrition or related illness arising from their unique dietary patterns, differing from others in the group. Preservation bias in the remains of adult compared to non-adults may also arise from differences in bone density [96]. Additionally, the frequency of certain foods in the archaeological record could reflect better preservation potential, for example, foods associated with animal bones or shell middens compared to eggs or plant-based foods. Though, our review included few studies drawing heavily on archeological records. Reporting bias may have been present in ethnographies observed to capture foods returned to camps such as game, while missing fruits and fish and seafoods foraged by women and children [97]. Our findings from both isotope studies as well as ethnographies supported the child consumption of aquatic animal foods in the form of small shellfish and animals found in shallow waters, pointing to the importance of this type of ASF.

We also acknowledge that the EA dietary pattern may not reflect diets today and some forms of malnutrition in children may arise from these differences, for example, the high consumption of ultra-processed foods. However, in our view elements of the EA dietary pattern offer vital insights relevant today with respect to lost dietary diversity and nutrient-poor, cereal-based diets. The granularity of climate data available through time was another potential limitation. And finally, the application of standardized food group categories may have narrowed our perspective on the full range of foods in child diets and masked other categories of nutritional importance (e.g. botanical features such as root, stem or leaves). Despite these limitations, this was a comprehensive review that applied robust methods in the search strategy, screening and interpretation processes.

## Conclusions

This research endeavored to fill an important gap in evidence for child nutrition during the evolution of *Homo sapiens*. Malnutrition affects large proportions of children today. This signals a likely disconnect between genome-derived biological nutritional needs and present dietary patterns. In our view, drawing insights from child dietary patterns of our evolutionary past might better inform public health solutions with greater impacts. Experimental trials are now needed to examine foods and dietary patterns identified here to better establish causal links to health outcomes. We acknowledge that modern diets cannot perfectly reflect those of our ancestors nor is this necessary to achieve health in populations. Replicating evolutionary diets will be challenging given ecological conditions today and the enormous complexity of global food systems. However, adapting certain elements of these dietary patterns may be especially critical for nutritionally vulnerable young children. In our view, we should work to ensure all children have equitable access to nutrient-dense foods and diverse diets more consistent with their evolutionary past.

## SUPPLEMENTARY DATA

Supplementary data is available at *EMPH* online.

## Supplementary Material

eoac027_Supplementary_Data

## Data Availability

The data underlying this article are available in in the online supplementary material.
