# Supplementary material for: Child dietary patterns in Homo sapiens evolution: A systematic review
Source: Evol Med Public Health. 2022 Jul 26;10(1):371–90. doi: 10.1093/emph/eoac027 (PMC9415195; doi:10.1093/emph/eoac027)
Supplement: eoac027_Supplementary_Data [file eoac027_supplementary_data.docx]

**SUPPLEMENTARY DATA**

**Supplementary Figure 1.** PRISMA Flow Diagram


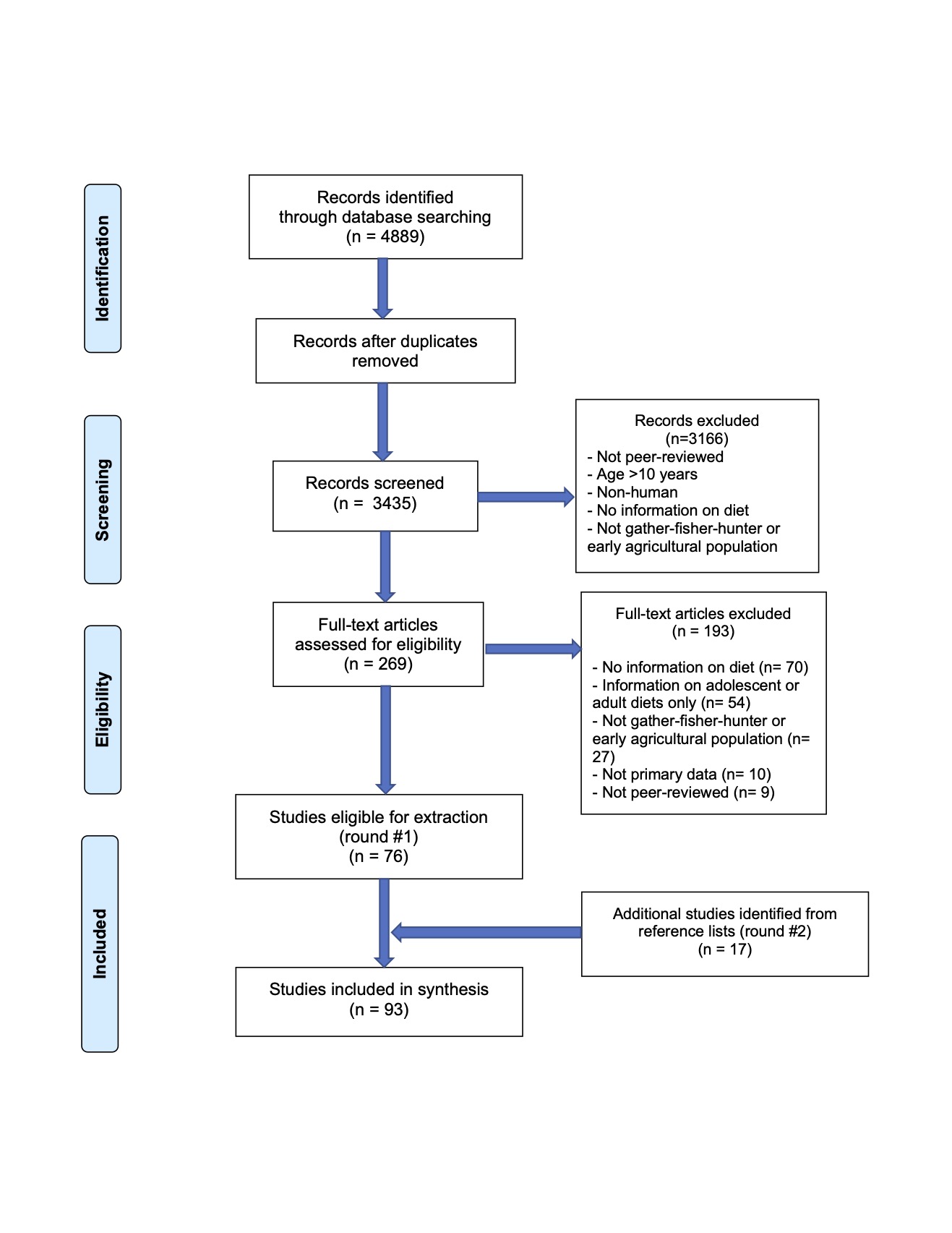


**Supplementary Table 1.** Search strategy by database

| **Academic Search Complete Via EBSCO** |
| --- |
| DE "INFANTS" OR DE “CHILDREN” OR child* OR infant*) AND (DE "HUNTING & gathering societies" OR “pre agricultural” OR “prehistoric” OR “hunter gatherer*” OR forager* OR "pre industrial" OR archeological OR archaeological OR paleolithic OR pleistocene OR neolithic OR mesolithic OR archaic OR “stone age” OR epipaleolithic OR “paleo Indian” OR “jeulmun pottery period” OR “Jomon period”) AND (DE "NUTRITION" OR DE “DIET” OR diet OR food OR “weaning foods” OR “complementary feeding” OR nutrition OR “pre mastication” OR provisioning OR breastfeeding) |
| **Anthropology Plus Via EBSCO** |
| (ZU "children" OR ZU "infants" OR child* OR infant*) AND (DE "Hunting and gathering societies" OR “pre agricultural” OR “prehistoric” OR “hunter gatherer*” OR forager* OR "pre industrial" OR archeological OR archaeological OR paleolithic OR pleistocene OR neolithic OR mesolithic OR archaic OR “stone age” OR epipaleolithic OR “paleo Indian” OR “jeulmun pottery period” OR “Jomon period”) AND (DE "Diet" OR DE "Food” OR DE "Nutrition" OR diet OR food OR “weaning foods” OR “complementary feeding” OR nutrition OR “pre mastication” OR provisioning OR breastfeeding) |
| **PubMed Medline** |
| ("Child"[Mesh] OR "Infant"[Mesh] OR child* OR infant*) AND (“pre agricultural” OR “prehistoric” OR “hunter gatherer*” OR forager* OR "pre industrial" OR archeological OR archaeological OR paleolithic OR pleistocene OR neolithic OR mesolithic OR archaic OR “stone age” OR epipaleolithic OR “paleo Indian” OR “jeulmun pottery period” OR “Jomon period”) AND ("Diet"[Mesh] OR "Diet, Food, and Nutrition"[Mesh] OR "Weaning"[Mesh] OR "Feeding Behavior"[Mesh] OR diet OR food OR “weaning foods” OR “complementary feeding” OR nutrition OR “pre mastication” OR provisioning OR breastfeeding) NOT (("Animals"[Mesh]) NOT ("Animals"[Mesh] AND "Humans"[Mesh])) |
| **Scopus** |
| TITLE-ABS-KEY(child* OR infant*)AND  TITLE-ABS-KEY(“pre agricultural” OR “prehistoric” OR “hunter gatherer*” OR forager* OR "pre industrial" OR archeological OR archaeological OR paleolithic OR pleistocene OR neolithic OR mesolithic OR archaic OR “stone age” OR epipaleolithic OR “paleo Indian” OR “jeulmun pottery period” OR “Jomon period”) AND TITLE-ABS-KEY(diet OR food OR “weaning foods” OR “complementary feeding” OR nutrition OR “pre mastication” OR provisioning OR breastfeeding)  AND KEY(human OR humans OR woman OR man OR women OR men OR child* OR adolescent* OR teen*) |
| **SocIndex with Full Text Via EBSCO** |
| (DE "Infants" OR DE "Children" OR child* OR infant*)  AND (DE "Hunting & gathering societies" OR “pre agricultural” OR “prehistoric” OR “hunter gatherer*” OR forager* OR "pre industrial" OR archeological OR archaeological OR paleolithic OR pleistocene OR neolithic OR mesolithic OR archaic OR “stone age” OR epipaleolithic OR “paleo Indian” OR “jeulmun pottery period” OR “Jomon period”) AND (DE "Diet" OR DE "Food” OR DE "Nutrition" OR diet OR food OR “weaning foods” OR “complementary feeding” OR nutrition OR “pre mastication” OR provisioning OR breastfeeding) |

**Supplementary Table *2*.** Data extraction summary

| **Reference** | **Study Design** | **Subsistence Method** | **Sample Size** | **Age** | **Time Period, Year** | **Place** | **World Bank Regions** | **Köppen-Geiger Climate Zone** | **Animal Source Foods** | **Plant Source Foods** | **Complementary foods** | **Food preparation** | **Sex-based differences in diet** | **Similarities/**  **Differences with adult diet** | **Ages for introduction of complementary foods (CF) and breastfeeding cessation (BC)** |
| --- | --- | --- | --- | --- | --- | --- | --- | --- | --- | --- | --- | --- | --- | --- | --- |
| Berbesque [1] | Analysis of non-specific stress markers | Gatherer-hunter | 119 | - | Early to Late Archaic Period, 2500-6500 BP | Buckeye Knoll site 41VT98 Victoria County, Texas | North America | C. Temperate | fish, rodents, deer, birds | maize, beans, squash | - | - | - | - | **CF**: Mean age=3.92 years |
| Bird [2] | Ethnography | Gatherer-hunter-fisher | 35 | 5-15 years | Modern History, 1993-1995; 1997-1998 | Great Barrier Reef, Papua New Guinea | East Asia & Pacific | A. Tropical | reef flat shellfish [Strombus luhuanus (black lipped conch), small Trochus niloticus (top-shell)] (These represent what children forage) | seasonal fruits and nuts, some types of tubers | - | - | - | Differences between adults and children in choices of prey; children forage lower-rank shellfish which require minimal field process and therefore conserve energy. | - |
| Bird [3] | Ethnography | Gatherer-hunter | 26 | 4-14 years | Modern History, 2000-2002 | Northwest of Western Desert, Australia | East Asia & Pacific | B. Arid | lizard, sand goana (Veranus gouldii), kangaroo, bustard, emu, goanna (Veranus acanthrus), blue-tongue skink (tiliqua scincoides), small birds, Cossid spp. Larvae, bird eggs | fruits--solanum diversiflorum, solanum ellipticum; wild onion, pencil yams, woolybutt grass | - | - | - | - | - |
| Bocherens [4] | Isotope Analysis | Gatherer-hunter-fisher-agriculture | 93 human bone fragments, with minimum number of child individuals ranging from 1- 13) | - | Mesolithic and Neolithic, 10000-2000 BC | Meuse Basin, Netherlands | Europe & Central Asia | C. Temperate | Ancient Mesolithic: dietary protein mainly provided by terrestrial mammals and freshwater resources; Middle Neolithic: Increased freshwater resources consumed due to deforestation limiting number of terrestrial food sources. Rest of protein came from terrestrial mammals, hunted wild mammals or domesticated mammals. | Increased consumption of plants (possibly cereals) in (late/middle) Neolithic populations. | - | - | - | - | **CF**: ~ 2 years;  Middle/Late Neolithic: before age 2;  Late Neolithic: Children were still breastfeeding at 1 year.  **BC:** Ancient Mesolithic: 2 years;  Middle/Late Neolithic: By age 2;  Late Neolithic: 2.5-3 years |
| Boyette [5] | Ethnography | Gatherer-hunter | 31 | 5-16 years | Modern History, 2008 | Southwestern Central African Republic, Northern Republic of the Congo. Ngandu village of Bagandou | Sub-Saharan Africa | A. Tropical | forest mice, caterpillars, duiker, honey, porcupine, cookie | manioc leaves, manioc; leaves of Gnetum sps, or seasonal foods, such as several varieties of nut, mushrooms, wild yams | - | - | Differences in foraging patterns: boys obtain more ASFs and females obtaining PSFs. However, there is food sharing between boys and girls. | - | - |
| Budd [6] | Isotope Analysis | Agriculture | 23 | 3 non-adults aged 3-12 years | Neolithic; Chalcolithic, 7000 BC to middle of 6000 BC | Aktopraklik site / Eastern Marmara region , Turkey | Europe & Central Asia | C. Temperate | Late neolithic: pig, sheep, goat. Neolithic: Sus domesticus, Bos taurus, Caprovine (sheep/goat), fallow deer.  Chalcolithic: herbivores (sheep/goat) | Chalcolithic: C3 terrestrial plants (not in large quantities) Neolithic: C3 based diet | - | - | - | One Chalcolithic sub-adult aged between 9 and 12 had isotopic ratios for carbon and nitrogen that were similar to that of Chalcolithic adult females, suggesting similarities between child and adult diets. A 3 year-old Neolithic child appears to have a similar diet to the adult as evidenced by similar isotope ratios. | **CF:** 3 years ±12 months in the Late Neolithic samples; 4 years ± 12 months for Early Chalcolithic |
| Bullington [7] | Analysis of non-specific stress markers | Agriculture | 36 | 6-27 months | Middle Woodland, 50 BC to 250 AD; Mississippian 1,000 AD to 1350 AD | Lower Illinois Valley | North America | C. Temperate | - | Horticultural (Middle Woodland): nuts and starchy seeds with tough seed coats; Agricultural (Mississippian): Maize, lower proportion of nuts (relative to horticultural group) | Complementary diets are more diverse for the horticultural sample and homogenous for the agricultural sample, where the diet was mostly maize processed in some form. | Agricultural groups boiled hard starch seeds. Generally, their diets were softer than those of Horticultural/Mid Woodland children. | - | Differences only pertain to texture, with adults consuming harder foods. | - |
| Castilla [8] | Analysis of non-specific stress markers | Gatherer-hunter | 1 sub-adult | approximately 6 years old | Chalcolithic, 5030 to 5020 BP | Sierra de Atapuerca, Spain | Europe & Central Asia | C. Temperate | domesticated animals and their derivatives | cereals of various types; inadequate intake of fruits and vegetables | - | - | - | - | **CF:** 1-3 years |
| Chinique de Armas [9] | Isotope Analysis | Gatherer-hunter-fisher | 21 non-adults | 0-10 years | Archaic Age, 390 -580 AD (Cueva del Perico); 480- 680 AD (Cueva Calero) | Cueva del Perico I and Cueva Calero, Cuba | Latin America & Caribbean | C. Temperate | Milk, meat, some marine/riverine resources | herbal teas, fruits, and root cultigens such as sweet potatoes, yautia (pap of ocumo), and arrowroots; wild plants | meat; milk; evidence of low protein, carbohydrate-rich foods: herbal teas, fruits, and root cultigens such as sweet potatoes, yautia (pap of ocumo), and arrowroots | Protein sources are premasticated | - | Statistically significant differences in carbon and nitrogen isotope ratios between all non-adult groups and adults. | **CF:** 9 months  **BC:** 5.5 years |
|  |  | Agriculture | 38 non-adults | 0-10 years | Ceramic Age, 1130±110 BC to 580±120 AD | Paso del Indio, Puerto Rico; Marco Gonzalez and San Pedro, Belize. | Latin America & Caribbean | C. Temperate | milk, C-13 enriched marine animals | herbal teas, fruits, and root cultigens such as sweet potatoes, yautia (pap of ocumo), and arrowroots; Mayan (Paso del Indio) - heavy maize consumption. | milk; evidence of low protein, carbohydrate-rich foods: herbal teas, fruits, and root cultigens such as sweet potatoes, yautia (pap of ocumo), and arrowroots; maize; terrestrial protein resources; C-13 enriched marine animals | - | - | Carbon isotope ratios are similar for sub-adults and adults, indicating similarity in diets. "Weaning food choices were made from available and typical adult foods." | **CF:** Higher probability at 2.7 years (Puerto Rico); Higher probability at 2.2 years (Belize)  **BC:** Higher probability at 5.9 years (Puerto Rico); Higher probability at 3.1 years (Belize) |
|  |  | Gatherer-hunter | 29 non-adults | 0-10 years | Archaic Age | Canimar Abajo, Cuba | Latin America & Caribbean | C. Temperate | milk | herbal teas, fruits, and root cultigens such as sweet potatoes, yautia (pap of ocumo), and arrowroots | milk; evidence of low protein, carbohydrate-rich foods: herbal teas, fruits, and root cultigens such as sweet potatoes, yautia (pap of ocumo), and arrowroots; legumes | - | - | Statistically significant differences in carbon and nitrogen isotope ratios between all non-adult groups and adults. | **CF:** Higher probability at 2 years  **BC:** Higher probability at 2.8 years |
| Chinique de Armas [10] | Isotope Analysis | Gatherer-hunter-fisher | 49 | 31 juveniles aged 0-6 years | 1130 ± 110 BC (at old cemetery); 580 ± 120 AD (at young cemetery) | Canimar, Abajo, Cuba (Matanzas City, Cuba). | Latin America & Caribbean | C. Temperate | - | starch-rich foods like root cultigens and legumes; tropical fruits. Legumes provide the bulk of dietary protein for children ages 2-6. | starch-rich foods like root cultigens and legumes, tropical fruits. Ipomoea batatas, Xantosoma sp., Phaseolus vulgaris, Zamia spp. | - | - | Statistically significant carbon isotope between Canímar Abajo adult females and each of the juvenile groups. Animal marine/riverine protein and maize are part of the diets of Canímar Abajo female adults' diets; however, these foods were likely not used to as part of the juveniles’ complementary feeding. | **CF:** Higher probability at ~ 1.7 years  **BC:** Higher probability at ~3 years |
| Choy [11] | Isotope analysis | Agriculture | 158 | 25 aged 0-10 | Three Kingdoms period, 300-600 AD | Busan City, South Korea | East Asia & Pacific | C. Temperate | freshwater fish, marine resources, sea lion, mullet, porgy | C3 terrestrial resources, C4 plants (foxtail millet), rice | C3-based supplementary foods. |  | Males consumed more protein or higher trophic level protein than females. | Non-significant changes in diet with age. | **CF:** 2 years  **BC:** 3-4 years |
| Clayton [12] | Isotope Analysis | Gatherer-hunter | 35 | 0-8.5 years | Later Stone Age, 12,000-7500 BP | Matjes River Rock Shelter, South Africa | Sub-Saharan Africa | B. Arid | fish, premasticated shellfish, and meat | C3 plant foods | C3 plant foods, fish, premasticated shellfish, and meat | shellfish is premasticated | - | - | **CF:** 1.5 years  **BC:** 2-4 years |
| Coltrain [13] | Isotope Analysis | Agriculture | 149 | 18 children aged 3-11 years | Basketmaker II, 415 BC - 322 AD | Four corners, USA | North America | D. Continental | Low intake of animal protein. Cotton tail rabbits | maize, yucca, wild C4 plant foods | maize gruel | maize made into gruel | - | Evidence suggests higher fat diets in infants (potentially because of breastmilk). Adult diets appear to be limited by the low fat content of their maize-dominated diets and low intake rates of lean animal protein. | - |
| Cook [14] | Analysis of non-specific stress markers | Gatherer-hunter-agriculture | 170 children | Under 6 | Middle Woodland, (100 BC - AD 500)/Late Woodland | Lower Illinois Valley | North America | C. Temperate | - | - | High carbohydrate, cariogenic foods | - | - | - | - |
| De Souza [15] | Ethnography | Gatherer-hunter | 253 participants | 109 under the age of 20 (defined as children in the study) | Modern History, 2002 | San Ildefonso Peninsula and Casiguran Area | East Asia & Pacific | A. Tropical | game (historically) | Historically (low-carbohydrate plants); Modern day: rice | Rice is often used as a substitute for breast milk. | - | - | - | - |
| Domínguez-Rodrigo [16] | Analysis of non-specific stress markers | Gatherer-hunter | 1 child | ~ 2 years old | Pleistocene, 1.5 Ma | Olduvai Gorge, Tanzania | Sub-Saharan Africa | B. Arid | meat | - | meat (very low amounts consumed by mother or by child depending on if the child was weaned or not) | - | - | - | **BC:** 2.5 years |
| Early [17] | Ethnography | gatherer-hunter-fisher | Demographic study--sample sizes are "hypothetical" | - | Modern History, 1976-1984 | Eastern Luzon, Phillipines | East Asia & Pacific | A. Tropical | monkey, deer meat, fish, wild pigs, canned milk (made of heavily sugared liquid from coconut or vegetable oil mixed with small amount of nonfat skim milk) | rice, sugar water | - | - | - | - | - |
| Eerkens [18] | Isotope Analysis | Gatherer-hunter | 6 | All individuals died as adults | Middle to Late Holocene, 4300-3100 BP | San Francisco Bay Estruary, USA | North America | C. Temperate | modest amounts of marine protein e.g. Shellfish and salmon; freshwater fish, snakes, turtles, frogs, lizards | acorns, C3 terrestrial vegetal foods | Acorn gruel; freshwater fish, snakes, turtles, and/or a range of smaller insectivorous species such as lizards or frogs | Acorns prepared into gruels | - | Two individuals in the sample had adult-like weaning foods (foods with higher trophic levels). One individual had low levels of marine derived proteins during the first 6 years of life, but consumption appears higher between the ages of 12 and 22. | **BC:** Average completion of weaning = 3.1-4.1years |
| Eerkens [19] | Isotope Analysis | Gatherer-hunter-fisher | 20 | All individuals died as adults | Middle Holocene, 4300-3000 BP | Central California, USA | North America | C. Temperate | marine-derived proteins; freshwater fish, terrestrial meat (esp. brain and liver) | Acorn or seed gruel | Carbohydrate-rich plant gruels; low-trophic level foods (eg. Gruel from acorns or seeds); fish, meat | Processing of acorns to form a gruel | Females consumed lower trophic-level foods than males. Girls consumed greater amounts of plants, and boys consumed greater amounts of higher-trophic level fish and meat protein. | Within this sample of adults, evidence suggests they consumed a different range of foods as adults and had more diverse diets as children, Children’s diets become similar to adults’ by age 7. | **CF:** Females-3.6 years; Males 3.2 years  **BC:** 5-6 years |
| Eerkens [20] | Isotope Analysis | Gatherer-hunter-fisher | 8 | All individuals died as adults | Middle Holocene, 3000-3300 BP | Current equivalent - city of Stockton in California | North America | C. Temperate | higher trophic level foods: fish , large game;reptiles, insects | plant-drived proteins | - | - | Boys and girls were eating similar types of foods and overall dietary composition was more homogenous among all children, suggesting a potential 'common pot' sharing pattern. | - | **CF:** Median of 1.7 years  **BC:** Median of 3.2 years |
| El-Najjar [21] | Analysis of non-specific stress markers | Agriculture | 539 Crania, 200 of which are from children | Children: 0-10 | Basketmaker II-IV (400-700 AD); Pueblo II-III (900-1300 AD); Pueblo III (1250 -1300 AD); Pueblo I-II (700-1100 AD); Pueblo IV-V (1315-1673 AD) | Canyon de Chelly, Chaco Canyon, inscription House, Navajo Reservoir, Gran Quivira. | North America | D. Continental | Deer, antelope, small game animals (Gran Quivira-Pueblo IV-V);  Pueblo I-II Navajo Resevoir - 10 species of fish, 41 animal species, 52 bird species  Basketmaker and Pueblo II-III populations have limited ASFs in their diet.) | Maize and beans eaten across all periods.  Basketmaker II-IV/ Pueblo II-III populations (predominantly maize diet, cactus fruit, pinon nuts, chenopods, squash, and beans, pumpkin.  Pueblo III populations (prdominantly maize, squash, gourds, prickley pear, bee weed) | - | - | - | - | - |
| Eriksson [22] | Isotope analysis | gatherer-hunter-fisher | 26 | 3 children | Middle Neolithic, 2900-2500 BC | Gotland, Sweden | Europe & Central Asia | D. Continental | Marine protein, seal, fish (not significant amounts) | Algae (limited amounts) | - | - | No sex differences in diet | Child diet was the same as adult diet. | - |
| Fernandes-Costa [23] | Ethnography | Gatherer-hunter | 217 subjects | 32 pre-pubertal children 6-14 years | Modern History, 1981 | Chum!kwe, Namibia | Sub-Saharan Africa | B. Arid | meat (Small to negligible parts of diet come from hunting activities and animal husbandry) | maize, vegetables, canned fruit, melons and squashes (negligible) | - | maize made into porridge or fermented with cane sugar to make alcoholic beverages; meat and maize porridge is boiled in aluminum pots (iron went out of favor) | - | - | **BC:** 4 years |
| Fernández-Crespo [24] | Isotope Analysis | Agriculture | 17 juveniles + additional data obtained from dental remains of 7 adults | The 7 died as adults; Juveniles: 4-7 years | Late Neolithic, 3500-2900 BC | Laguardia, Alava | Europe & Central Asia | C. Temperate | dairy products and meat (sheep, pig, cattle, goat) | C3 Cereals | dairy products and meat | C3 cereals prepared as gruels | No statistical differences in older children's diets, but female infants appear to have been exclusively breastfed for longer than males i.e boys receive complementary foods earlier | Fully weaned children have similar isotope values to adults, indicating that their post-weaning diet is supplemented by foods provisioned by parents. However, around ages 9-11, Carbon and nitrogen Isotope values decreased in 3 female individuals. This is possibly attributed to dietary restrictions to prepare them for adulthood. | **CF:** Mean = ≤1.1 years  **BF:** 3.9 years |
| Fernández-Crespo [25] | Isotope Analysis | Agriculture | 70 | 5 "older children" and 3 "younger children" (ages not specified) | Middle Chalcolithic, 2900-2500 BC | mid-upper Ebro Valley, Spain | Europe & Central Asia | C. Temperate | Terrestrial Animal Sources: meat from both domestic and wild sources (eg. Wild pig), dairy products from cattle/sheep/ goat. | C3 Plants: wheat, barley; forest fruits and nuts (e.g.apples, pears, wild berries, hazelnuts, acorns) | - | - | - | Notable differences in Carbon and Nitrogen Isotope ratios between non-adults and adults, with Nitrogen values increasing with age. May be attributed to higher protein intake among adults or physiological processes (eg. Skeletal growth), or juveniles having compromised health. | - |
| Fontanals-Coll [26] | Isotope Analysis | Gatherer-hunter-fisher-agriculture | 25 | 4 sub-adults aged 6-11 years | Neolithic, late 5^th^ to 4^th^ millenia BC | North-East Iberian Penninsula, Europe | Europe & Central Asia | C. Temperate | Possible occasional consumption of freshwater resources; dairy, possible occasional consumption of meat obtained from livestock. Ovicaprines (sheep/goat), cattle, swine and occasionally wild animals. | C3 terrestrial resources: wheat, barley; Legumes: fava beans, lentils and peas. No evidence of C4 plants. | - | - | - | Based on similarities between sub-adult and adult Nitrogen and Carbon isotope ratios, it is inferred that diets are similar to adults' at ages 6 - 11. | - |
| Fouts [27] | Ethnography | Gatherer-hunter-agriculture | 22 forager children; 21 farmer children | 18-59 months | Modern History, 1998-2001 | Ngotto Forest (Congo Basin), Central African Republic | Sub-Saharan Africa | A. Tropical | Bofi Foragers: duikers, rats, porcupines, mongoose, hogs, monkeys, honey, insects  Bofi Farmers: insects | Bofi foragers: leaves, mushrooms, roots, nuts, fruits,   Bofi Farmers: manioc, coffee, corn, peanuts, a variety of fruits, mushrooms, leaves | Farmers: rice gruels/rice, foragers - no specially prepared foods for weaning age children. | - | - | - | **BC:** Foragers: 36 - 53 months  Farmers: 18 -27 months |
| Fouts [28] | Ethnography | Gatherer-hunter | 12 | 18-59 months | Modern History | Ngotto Forest (Congo Basin), Central African Republic | Sub-Saharan Africa | A. Tropical | duikers, monkeys, rats, porcupines, mongoose, hogs, caterpillars, insects | mushrooms, leaves, nuts, fruits, roots | Percentages indicate frequency of observation with each food: manioc (37%), wild yams (18%), green leafy vegetables (12%), caterpillars (12%), nuts (7%), corn (4%), bush meat (4%), fruits (3%), snails (3%), and mushrooms (1%), rice mashes. | - | - | - | **BC:** 3+ years or when the mother is pregnant |
| Fouts [29] | Ethnography | Gatherer-hunter | 22 | 2-4 years (Mean age = 42.7 months) | Modern History, 2004-2005 | Northwest Republic of Congo, Likouala region | Sub-Saharan Africa | A. Tropical | duiker, insects, rats, porcupines, mongoose, and monkeys, meat, fish, caterpillars, insects, honey | fruits, vegetables, green leafy vegetables, palm oil/palm fruit, and nuts,mushrooms and roots--yams, manioc, plantains. | Wild foods: yams, fruits, nuts, meat, fish, green leafy vegetables, caterpillars, and honey. Cultivated foods manioc, plantains, fruits, green leafy vegetables, palm oil (e.g., chewing on roasted palm nut fibers to consume oil), and nuts. | - | - | Children begin to eat adult food starting from age 2. | **BC**: 3-4 years |
| Gardner [30] | Isotope Analysis | Gatherer-hunter-fisher | 201 | 22 children aged 0-10 years | 780-230 BP | Santa Clara County, California | North America | C. Temperate | elk, deer, rabbits, fish, sea otters, marine mammals, | kelp, acorns, tubers roots and rhizomes | higher trophic level proteins (freshwater fish and marine food), acorn soup, premasticated meat, pinole (seed meal made into mush cakes) | Meat: premastication; Acorn: Made into soups; Seeds: Made into mush cakes | - | Between the ages of 3 and 5, the nitrogen isotope values are slightly higher compared to adult females', reflecting increased supplementation with complementary foods. By ages 6-10, nitrogen isotope values become lower than that of the adult females, suggesting complete weaning and different dietary patterns from adults. | **CF:** 0.8 years  **BC:** 4.4 years |
| Garralda [31] | Analysis of non-specific stress markers | Gatherer-hunter | 1 child | 4-5 years | Upper Paleolithic, 29,300 – 28,300 cal  BP | Cantabria, Northern Spain | Europe & Central Asia | C. Temperate | marine protein, terrestrial resources (horses and deer) | - | - | - | - | - | - |
| Goodman [32] | Analysis of non-specific stress markers | Gatherer-hunter | 111 adults and adolescents (48= late Woodland/Missippian acculurate Late Woodland horizons); 61 = Middle Mississippian | All individuals died as adolescents or adults | Late Woodland/ Mississppian Acculturated Late Woodland (AD 950-1150); Middle Mississippi (AD 1150-1300) | Dickson Mounds, Lewistown, Illinois | North America | D. Continental | - | maize | maize | - | - | - | **CF:** 1.5 years  **BC:** 2-4 years |
| Greenwald [33] | Isotope Analysis | Gatherer-hunter | 24 | 9 juveniles aged 7-18 | Late Holocene, 1100-700 BP | Pleasanton, California | North America | C. Temperate | Waterfowl, rodents, fish, shellfish | Acorns; small seeds—hairgrass (*Deschampsia* spp.), fescue (*Vulpia* spp.), clover (*Trifolium* spp.), farewell to spring (*Clarkia* spp.), red maids (*Calandrinia* spp.), and goosefoot (*Chenopodium* spp.)  Flora—geophytes (e.g. Brodiaea), bay (Umbellularia californica), buckeye (Aesculus californica), and hazel (Corylus cornuta var. californica), fiddleneck (Amsinckia spp.), saltbush (Atriplex spp.), brome grass (Bromus spp.), miners lettuce (Claytonia spp.), elderberry (Sambucus mexicana), tarweed (Madia spp.), maygrass (Phalaris spp.), and dock (Rumex spp.) | - | proteins are pounded | - | Similarities between immediate post-weaning diets and adult diets; findings suggest that adults and weanlings derive dietary proteins from similar sources (similar N-15 levels)..This is a result of adults provisioning children. However, after the post-weaning period where there are similarities between parental and child diets, there are differences in diets, with levels of N-15 dropping markedly during middle childhood, indicating that children consumed different resources from adults due to independent foraging behaviors. Children possibly consumed foods from lower trophic levels including plants which require minimal processing and small vertebrates. | **CF:** 11.6 months (Mean)  **BC**: 28 months or 2.3 years |
| Halcrow [34] | Analysis of non-specific stress markers | Agriculture | - | Mean ages across different sites range from 3.3 years to 5.9 years | Neolithic to Iron Age, 4000 to 1500 BP | Thailand and Cambodia, South East Asia | East Asia & Pacific | A. Tropical | - | rice, wild and cultivated taro and yams | wild and cultivated taro and yams, rice | wild and cultivated taro and yams are mashed | - | - | **BC:** 2-3 years |
| Han [35] | Other | Gatherer-hunter-fisher | 12 infant-mother dyads | 0.8-2 years | Modern History | Bolivian Amazon, Bolivia | Latin America & Caribbean | A. Tropical | foraged game, fish, meat | plantain, vegetable oils, rice | stews made from roasted plantain and meats and fish. | Premastication through the first two years of life, particularly for foods that are too hot, too dry or pose choking hazards (eg. roasted plantains, stews, meat, and fish.  Stews made from plantain and meat: "boiling approximately 1 kg of dried beef, 20 unripe plantains, and vegetable oil and salt to taste." | - | - | **CF:** 4 months  **BC:** 19 months |
| Hawkes [36] | Ethnography | Gatherer-hunter | 35 -50 | NA | Modern History, mid/late 1980s | Lake Eyasi (Northern Tanzania) | Sub-Saharan Africa | A. Tropical | meat (from about 43 large animals), honey; Average acquisition rate for daytime big game in forager's households: 120 Calories/forager-hour | Baobab; Average acquisition rate for PSFs in forager's households: > 1000 Calories/forager-hour | - | - | - | - | - |
| Hawkes [37] | Ethnography | Gatherer-hunter | 90 | 33 children: weaned - 13 years | Modern History,1985-1986 | Lake Eyasi (Northern Tanzania), Tli'ika district. | Sub-Saharan Africa | A. Tropical | honey; meat (large carcasses--impala, giraffe, zebra, elephant, eland) | Vigna frutescens (tuber); Baobab; Berries, | - | - | - | - | - |
| Hawkes [38] | Ethnography | Gatherer-hunter | 48 persons | 10 girls and 15 boys: Mean age for girls: 8.4 years (3.5-13.6); Mean age for boys: 11.5 years (5-17) | Modern History | Lake Eyasi (Northern Tanzania), Tli'ika district. | Sub-Saharan Africa | A. Tropical | honey | tamarind fruit, berries, tubers, baobab fruits. Baobab fruits are estimated to provide 5-10 year olds about an average of 1,300 calories/ hour of foraging and 2,100 calories/ hour of foraging to 11-15 year olds. | - | - | - | - | - |
| Herrscher [39] | Isotope Analysis | Gatherer-hunter-agriculture | 57 | 19 sub-adults | Middle Neolithic, 4500-3500 BC | Toulouse, France | Europe & Central Asia | C. Temperate | Freshwater fish, eel (marine and freshwater not a major part of the diet), meat and/or dairy products | cereals | - | - | - | Child diet is the same as adult diet. |  |
| Hirasawa [40] | Ethnography | Gatherer-hunter-fisher-agriculture | 23 | Mean: 8.1 months | Modern History, 2000-2001 | Mbeson, Cameroon | Sub-Saharan Africa | A. Tropical | - | ripe plantain, wild yam, hot pepper sauce | ripe plantain, wild yam, hot pepper sauce | plantain and yam are mashed | - | Child diet is the same as adult diet. | **CF:** 6 months  **BC:** When mother has next pregnancy; approximate birth interval is 35 months. |
| Howcroft [41] | Isotope Analysis | Gatherer-hunter-fisher | 42 | 24 sub-adults | Middle Neolithic, 3000-2750 BC | Gotland, Sweden | Europe & Central Asia | D. Continental | seal products (ringed seal, harp seal); herring, cod, pig | - | fish-based products | - | Enamel hypoplasia was more prevalent in males. This suggests that an individual’s “sex was one determinant of dietary variability in the post-weaning period, with female sub adults consuming a diet that offered greater protection against environmental stresses.” | Complementary foods are obtained from similar sources as the adult diet, i.e. around 6 months. The infant and sub-adult diet is similar to the adult diet, which is rich in seal products. | **CF:** 6-11 months  BC: 3-4 years |
| Itahashi [42] | Isotope Analysis | Gatherer-hunter | 15 | 4 juveniles/sub-adults aged 0-8 | Neolithic, 10^th^ Millenium BC | Batman, Turkey | Europe & Central Asia | D. Continental | goats, sheep, boars, freshwater fish, waterfowl | broad beans, peas, wild nuts | - | - | - | - | - |
| Itahashi [43] | Isotope Analysis | Agriculture | 29 | 8 children | Pottery Neolithic, 6100 BC - 5950 BC | Upper Tigris Valley, Anatolia | Europe & Central Asia | D. Continental | little cattle, limited amount of freshwater resources (aquatic protein), half of their protein from terrestrial animals (pigs, sheep, goat, cattle) | little or no C4 plants--chickpeas, lentil)  C3 plants such as wheat, barley, and pistachio | - | - | - | Child diet is the same as adult diet. |  |
| Ivey [44] | Ethnography | Gatherer-hunter-agriculture | 20 | 12-15 months | Modern History, 1988-1989 | Ituri, DR Congo | Sub-Saharan Africa | A. Tropical | meat, honey, fish, crabs, lean forest game | Diet is comprised of 60% carbohydrates: bananas, cassava, hot pepper paste, mushrooms, peanuts, rice, palm nuts, opi fruit, vegetables, wild yam | bananas, cassava, meat, hot pepper paste | cassava and bananas are mashed | - | - | **CF:** 2-3 months for mashed foods; 5-6 months for solid foods  **BC:** 3 years |
| Kamei [45] | Ethnography | Gatherer-hunter-fisher | 34 | 4-15 years | Modern History, 1997-1998 | East Province, Cameroon | Sub-Saharan Africa | A. Tropical | lizard, mouse, termites, insects, fish, chicken | papaya, plantains, cassava, sweet potato, grapefruit, maize, yaoutia | - | - | - | - | - |
| Katzenberg [46] | Isotope Analysis | Gatherer-hunter-agriculture | 29 | 14 (ages 0-10) | 1530-1580 AD | Hamilton, Ontario, Canada | North America | D. Continental | - | Maize | High carbohydrate (maize) weaning diet | If a mother died before a child was weaned, the father would feed it water in which corn has been boiled. | - | - | - |
| Keller [47] | Isotope Analysis | Gatherer-hunter | 9 | 6 children: neonatal age-6 years | Late Neolithic, 4250-3650 BC | Untergrombach, Germany | Europe & Central Asia | D. Continental | meat, freshwater fish, domestic animals | vegetables | - | - | - | Children consumed lower amounts of vegetable foods than adults. | - |
| Kinaston [48] | Isotope Analysis | Gatherer-hunter-fisher-agriculture | 27 | 12 sub-adults (age 0-5) | Initial Lapita, 2800–2600 BP); Later Lapita, 2600–2500 BP; Post-Lapita, 2500–2000 BP | Uripiv, Vanuatu | East Asia & Pacific | A. Tropical | Fish, shellfish, crustaceans, bats, birds, turtles | Taro, banana, yams, likely unspecified fruits and nuts | - | - | - | Post-weaning diets had higher nitrogen values, indicating that children consumed higher amounts of marine protein. Authors suggested that children may have collected and consumed mangrove and marine organisms as a snacks throughout the day. | - |
| Kinaston [49] | Isotope Analysis | Gatherer-hunter-fisher-agriculture | 142 | 30 children ages.1-9 years | 700-300 BP | Taumako, Solomon Islands | East Asia & Pacific | A. Tropical | Fish, shellfish, crustaceans, bats, birds, turtles, insects | starchy root vegetables, nuts, fruits, seaweed | - | - | Not in children, but in adults | Post-weaning child diet comprised slightly more terrestrial food and less marine and,  possibly, less C4 plants than in their later lives. In general, lower trophic protein sources. | - |
| King [50] | Isotope Analysis | Gatherer-hunter-fisher-agriculture | 1 | 5 years | Alto Ramírez phase of the Formative Period, 500 BC–AD 200 | Atacama Desert, Chile | Latin America & Caribbean | B. Arid | - | Maize | Maize gruel | Maize gruel | - | Isotopic evidence suggests that the child was weaned almost solely on maize. However, evidence on adults studied from that period indicates that marine resources were a significant part of their diets. | **CF:** 0.5 years  **BC:** 3 years |
| King [51] | Isotope Analysis | Gatherer-hunter-fisher-agriculture | 39 | 26 children aged 1.5-9 years | Archaic to Late Formative Period, 4000 BC- 450 AD | Atacama Desert, Chile | Latin America & Caribbean | B. Arid | - | Maize, Tubers | Maize gruel, tubers | - | - | Diets were more plant-based with some children in the sample. | **BC:** 1.5-3.5 years |
| Kramer [52] | Ethnography | Gatherer-hunter-fisher | 235 | NA | Modern History, 2006-2007 | Southwest Venezuela | Latin America & Caribbean | A. Tropical | Fish, small game | Roots, mango, manioc | - | - | - | - | - |
| Lukacs [53] | Analysis of Non-specific Stress Markers | Gatherer-hunter | 47 | 1 child aged 3-3.5 years | Mesolithic, 8800-8600 BC | Damdama, India | South Asia | C. Temperate | - | wild plants including fruit | - | Minimal preparation, food was very tough and fibrous | - | Child diet is the same as adult diet. | - |
| Lynnerup [54] | Other | Gatherer-hunter-fisher | 14 | 7 children aged .75-4.5 years | Prehistoric, 1200-1700 AD | Qilakitsoq, Greenland | Europe & Central Asia | E. Polar | Marine mammals, fish, reindeer, walrus | Mosses, other plants | Mostly meat, some plants | Children as young as 18 months were fed raw meat | - | - | - |
| Macchiarelli [55] | Isotope Analysis | Gatherer-hunter-fisher | 49 | 20.46% aged 0-5 | 5th-4th Millenium BC | Qurum, Oman | Middle East & North Africa | B. Arid | molluscs, fish, marine mammals, turtles | - | - | - | - | - | - |
| Marsteller [56] | Isotope Analysis | Gatherer-hunter-fisher-agriculture | 113 | 24 Infants and children | Late Intermediate Period, 900-1470 AD | Rimac Valley, Peru | Latin America & Caribbean | B. Arid | marine protein | mix of C3 and C4 plants, examples of potential C4 plants include maize, kiwicha, cati, and succulents | marine protein and mix of C3/C4 plants | - | - | In general, both children and adults ate a diet that was a mix of C3/C4 plants and 100% marine protein. However, 1/3 of adults/adolescents had less marine protein than the norm, while only 1 of the 24 children did. | - |
| Martin [57] | Ethnography | Gatherer-hunter-fisher-agriculture | 161 | 0-3 years | Modern History, 2012-2013 | Beni Department, Bolivia | Latin America & Caribbean | A. Tropical | Meat, fish, broth | rice, plantain, pasta, chincha (fermented manioc, corn, or plantain drink) | Meat, fish, broth, rice, plantain, pasta, chincha | “Most meals are “jo'na”, referring to any rice, plantain, or pasta-based stew mixed with meat or fish."  Foods that would be too difficult to chew are premasticated | - | Child diet is the same as adult diet. | **CF:** mean 0.32 years (reported) or 0.37 years (by survival analysis)  **BC:** mean 1.6 years (reported) or 2.25 years (by survival analysis) |
| Meehan [58] | Ethnography | Gatherer-hunter-agriculture | 93 mothers; 105 children | 0-4 years | Modern History, 2009-2012 | Congo Basin, Central African Republic | Sub-Saharan Africa | A. Tropical | Meat broth, honey, fish soups | Corn, banana, rice porridge, vegetables, biscuits | Meat broth, honey, corn, banana, soft-boiled vegetables, rice porridge | Vegetables are soft-boiled and not introduced until after 6 months, corn, banana, rice porridge, and meat broth are started between 1-3 months. | - | - | **CF:**  0-0.33 years; mean: ~ 0.25 years |
| Miller [59] | Isotope Analysis | Agriculture | 23 | Child diets inferred from adults. | Eastern Zhou, 771BC-221 BC | Xinzheng, China | East Asia & Pacific | D. Continental | pig, cattle/ox, dog, horse, sheep | Millet, soybean, wheat, | wheat and soybeans | - | Females were weaned earlier than males. | Early childhood diets show significant incorporation of C3 foods (wheat/soybean). Later adult diets have more millet, particularly for males. | **BC:** 2.5-4 years |
| Münster [60] | Isotope Analysis | Agriculture | 466 | 135 sub-adults aged 0-14 yrs | Mesolithic to Bronze Age, 5500-1550 BC | Middle Elbe-Saale region, Germany | Europe & Central Asia | D. Continental | Animal protein | - | - | - | - | Adults and adolescents ate relatively more animal protein than children. Starting around age 7, animal protein intake increased steadily until adulthood. | **CF:** 2.5 years  **BC:** 4 years |
| Oelze [61] | Isotope Analysis | Agriculture | 97 | 28 sub-adults aged 0-14 yrs | Neolithic, 5200-4800 BC | Saxony-Anhalt, Germany | Europe & Central Asia | D. Continental | Cattle, sheep, goat | C3 plants | - | - | No sex differences | Child diet is the same as adult diet. | **BC:** 3 years |
| Ogrinc [62] | Isotope Analysis | Agriculture | 25 | 13 children aged 1-12 yrs | Neolithic, 6400-5300 BP | Ajdovska jama, Slovenia | Europe & Central Asia | C. Temperate | meat , milk, domestic animals | cereals | - | - | - | Children age 5-12 had diet similar to adults, which was 44% domestic animals and 39% cereals | **CF:** 2 years |
| Pearson [63] | Isotope Analysis | Gatherer-hunter-fisher-agriculture | 145 | - (includes juveiles under the age of 20 years) | Neolithic, mid-8th millennium to the end of the 7th  millennium cal. BC | Çatalhöyük, Turkey | Europe & Central Asia | C. Temperate | Cattle, sheep, wild boar, hare, freshwater fish, birds and bird eggs, reptiles such as tortoise or mollusks. | glume wheat, bread wheat, naked barley, pea, lentil, bitter vetch, wild mustard, almond, acorn, pistachio, hackberry and fig | - | - | No sex differences | Plant only diet in children 5-10 years old | **CF:** 1.5 years  **BC:** 3 years |
| Pearson [64] | Isotope Analysis | Gatherer-hunter-fisher-agriculture | 20 | 0-5 yrs | Neolithic, mid-9th to the mid-8th millennium | Aşıklı Höyük and Çayönü Tepesi, Turkey | Europe & Central Asia | C. Temperate | wild boar, ruminants | Pulses, cereals | Pulses, cereals | - | - | Children are weaned on a solely plant-based diet | **CF:** 1-2 years  **BC:** 2-3.5 years |
| Scharlotta [65] | Isotope Analysis | Gatherer-hunter | 28 | All individuals died as adults | Early Neolithic, 7000–  5700 BP | Lake Baikal, Siberia | Europe & Central Asia | E. Polar | fish/other animal gruels | cereal gruels | fish and cereal gruels | Foods are softened/pre-chewed by adults | No sex differences | Similarities in in measures for carbon and nitrogen isotopes for adults and children suggest similar diets. However, carbon sources may differ between mother and child depending on trophic levels consumed; there may be differences in the fish species that individuals consume as children and those they consume as adults. | **CF:** 0.7-1.5 years  **BC:** 1.5-6.5 years |
| Schniter [66] | Ethnography | gatherer-hunter-agriculture | 421 | 9 individuals ages < 8 | Modern History , 2002-2006 | Beni Department, Bolivia | Latin America & Caribbean | A. Tropical | hunted game, wild caught freshwater fish, domesticated free-range poultry and live- stock | collected fruits and nuts – both wild and cultivated (6%), swidden horticulture –primarily rice, manioc, and corn (66%), and only minimally supplemented with market goods such as purchased crackers, salt, sugar, pasta, and cooking oil (2%) | - | - | - | - | - |
| Schurr [67] | Isotope Analysis | Gatherer-hunter | 33 | 0-6 years | Late Archaic, 5000-3500 BC | Ohio Valley, North America | North America | D. Continental | freshwater aquatic foods (aquatic-rich summer foods depleted in C-13) | Terrestrial foods (C3 plants) | aquatic-rich summer foods depleted in C-13 | - | - | Collagen isotope ratios of infants <1 year are possibly an indication of seasonal differences in diets that are not visible in adults "because of the longer turnover time of collagen for older individuals."  Yet, children's diets become similar to mothers' when they reach age 5. | **CF:** Likely before 1 year  **BC:** Weaning completed by age 5. |
| Schurr [68] | Isotope Analysis | Agriculture | 281 | 120 (age foetal to 14 years old) | Late Archaic, 3500-1000 BC; Mid-Missippian, 1300 - 1450 AD | Vanderburgh County, Indiana USA | North America | C. Temperate | Aquatic resources | Maize | - | - | - | - | **BC:** 3 years |
| Sealy [69] | Isotope Analysis | Gatherer-hunter | 3 | 2-7 years | Late Stone Age, 2145 ± 50 BP; 1985 ± 50 BP | Western Cape, South Africa | Sub-Saharan Africa | B. Arid | terrestrial proteins | - | - | - | - | Child diet is the same as adult diet. | - |
| Sealy [70] | Isotope Analysis | Gatherer-hunter | 122 | 29 juveniles ages 0-9 | Holocene, 10,000-1900 BP | Cape Province, South Africa | Sub-Saharan Africa | B. Arid | marine foods (shellfish, meat of whales, seals, fish, seabirds); meat of small bovids, tortoises, hyrax; C4-based terrestrial food (meat of grazing anmals) | starchy underground corms | - | - | - | - | - |
| Shack [71] | Ethnography | Gatherer-hunter | - | - | Modern History | East Sepik Province, Papua New Guinea | East Asia & Pacific | A. Tropical | Fish, chicken, tinned meat | Coconut, Sago, Green leaves, yams, taro, bananas, sweet potatoes, cassava, pawpaw, oil, [sweets], rice | - | Sago eaten as pancake or jelly like substance | - | - | - |
| Shuler [72] | Analysis of Non-specific Stress Markers | Agriculture | 1131 | Unspecified Number of children | Early Missippian, 500-1200 AD | Tombigbee Valley, Alabama/Mississippi, USA | North America | C. Temperate | - | Maize | Maize gruel | - | - | - | - |
| Smith [73] | Isotope Analysis | Gatherer-hunter-fisher | 22 | 0-10 years | Formative Period, 1500 BC- AD 400 | Atacama Desert, Chile | Latin America & Caribbean | B. Arid | High trophic level marine proteins (high fat, high protein raw fish) | - | High trophic level marine proteins (high fat, high protein raw fish) | Fish is consumed raw | - | - | **CF:** 0.2 years  **BC:** 2.5 years |
| Stantis [74] | Isotope Analysis | Gatherer-hunter-fisher-agriculture | 25 | 1 sub-adult | Vuda Phase, 750-150 BP | Viti Levu, Fiji | East Asia & Pacific | A. Tropical | shellfish, fish | Nuts, taro, yams | - | - | - | Children consumed more nuts and shellfish than adults; may be due to age- based diets or snacking while foraging | - |
| Stefanović [75] | Other | Agriculture | - | - | Neolithic, 5800−5450 cal BC | Grad-Starčevo, Serbia | Europe & Central Asia | D. Continental | animal milk | cereals (gruels) | - | cereals prepared as porridge | - | - |  |
| Szczepanek [76] | Isotope Analysis | Agriculture | 26 | 6 sub-adults aged 1-7 years | Late Neolithic, 2900-2300 BP | Malopolska Upland, Poland | Europe & Central Asia | D. Continental | herbivore meat, dairy protein | C3-based terrestrial plants | - | - | - | Children ate less animal protein than adults. | **BC:** 4 years |
| Tessone [77] | Isotope Analysis | Gatherer-hunter | 51 | 24 children aged 0-13 yrs | Late Holocene, 800 BP to 350 BP | Patagonia (Lago Salitroso), Argentina | Latin America & Caribbean | E. Polar | Guanaco fat/meat, choique fat/meat | Fruits and starchy vegetables (potentially yucon) | mammal and bird fat/meat, fruits, starchy vegetables | - | - | - | **CF:** 0.75-2 years  **BC:** 5-6 years |
| Townsend [78] | Ethnography | Gatherer-hunter-fisher | 25 women who have completed their reproductive cycle | - | Modern History | Wogamus River, New Guinea | East Asia & Pacific | A. Tropical | meat from feral and domesticated pigs, small game, fish, insect larvae | sago palm, bananas, starchy tubers, breadfruit, seeds, pandanus fruit, leafy greens | bananas, larvae of sago beetles, sago, meat | sago is prepared dry, crumbly or roasted. It's starrch is extracted from the trunk. | - | - | - |
| Trancho [79] | Isotope Analysis | Gatherer-hunter-fisher | 1 | 8 yrs | Mesolithic, 7930-7600 BP | Sheikh Mustafa, Central Sudan | Sub-Saharan Africa | B. Arid | Fish, Milk | Tubers, Vegetables, sorghum, setaria | - | - | - | - | - |
| Tsutaya [80] | Isotope Analysis | Gatherer-hunter-fisher | 58 | 0-15 yrs | Okhotsk Culture, 5^th^ -13^th^ century AD | Hokkaido, Japan | East Asia & Pacific | D. Continental | Marine fish, fats | Terrestrial foods (C3 plants) | fish, fish fat, C3 terrestrial plants | Pre-mastication | - | Adults consumed more marine mammals, while child diet was mostly fish, fish fat/liver, and terrestial C3 foods | **CF**: 0.4 years  **BC:** 1.8 years |
| Tsutaya [81] | Isotope Analysis | Gatherer-hunter-fisher | 41 | 0-14 yrs | Epi-Jomon Period, 2300-1700 years BP | Hokkaido, Japan | East Asia & Pacific | D. Continental | Roe, marine fish, marine mammals, terrestrial mammals | Tubers | One group weaned on marine fish/mammal diet, similar to adults. Another weaned on a diet that indicated some non-marine milk substitute, potentially some combination of fish roe, tubers and terrestrial mammals | Milk substitute consisting of mashed herring roe, cut cow parsnip root, boiled lily root, and seal oil.  Foods such as salmon, or ribs of bear, hare, or dog are premasticated for sub-adults. | - | One group in the study was weaned on an adult diet and another group was weaned on a different diet and then introduced an adult diet postweaning. | **BC:** 4-6 years |
| Tsutaya [82] | Isotope Analysis | Gatherer-hunter | 86 | 39 sub-adults aged 0-15 years | Late/Final Jomon,  4000 -2300 years BP | Atsumi Penninsula, Aichi Prefecture, Tokai region, Eastern Japan | East Asia & Pacific | C. Temperate | Marine animal products (fish and shellfish) Terrestrial mammals | Terrestrial foods (C3 plants) | Marine animal products (fish and shellfish) and terrestrial (C3 plants and mammals) | - | - | Child diet is the same as adult diet. | CF: 0.3 – 3.5 years |
| Tucker [83] | Ethnography | Gatherer-hunter | NA | - | Modern History | Mikea Forest, Madagascar | Sub-Saharan Africa | A. Tropical | - | wild watermelon, ovy (Dioscorea acuminata-wild yam), babo (Dioscorea bemandry), ba (unknown scientific name) | - | - | - | - | - |
| Ungar [84] | Analysis of non-specific stress markers | Gatherer-hunter | 76 | Child diet information obtained from adult data. | Modern History, 2015 | Lake Eyasi, Northern Tanzania | Sub-Saharan Africa | A. Tropical | Bush diet: honey, bee larvae, game, meat, birds (13-18% tubers, 4-18% baobab fruit, 18-22% beries figs drupes and legumes, 11-14% honey/larvae, 31-32% meat, 1-8% ag products, based on other studies) Village diet: domesticated meat | Bush diet: tubers, baobab fruit/flour/, Transient diet: Berries, fruit, palm fruit, baobab fruit.  Village diet: maize and sorghum, beans, rice, wild herbs (leafy greens). | Bush diet: tubers, baobab fruit/flour, honey, bee larvae, wild game meat, birds Village diet: maize and sorghum, rice, beans, domesticated meat | Village: meat (premasticated or broth), and cereals prepared as porridge; Bush: baobab flour as pre-masticated or as liquid (with water or berry juice); meat--pre-masticated or as broth; | No sex-based differences | - | - |
| Valentin [85] | Isotope Analysis | Gatherer-hunter-fisher-agriculture | 8 | 1 child aged 8-9 yrs | Historic Period, 1850 AD | Cikobia Island, Fiji | East Asia & Pacific | C. Temperate | Fish, Turtles (ASFs make up ~25% of the diet) | taro species, yams, sweet potato, pandanus, cordyline, breadfruit, coconut and kava, sugar cane  (PSFs make up ~75% pf the diet) | - | - | - | Child diet is the same as adult diet. | - |
| Veile [86] | Ethnography | Gatherer-hunter-fisher-agriculture | 312 infants | 0-36 months | Modern History, 2002-2007 | Beni Department, Bolivia | Latin America & Caribbean | A. Tropical | meat, fish | rice, plantain | watery stews of plantain or rice, meat or fish | watery stew of plantain or rice | - | - | **CF:** Of the infants <6mo, 28 started complementary feeding before 6mo and the average age of CF introduction was 3 months. Of all non exclusive BF infants (n=183), mean age of CF was 2.1 mo.  **BC:** Average age - 19.2 mo |
| Wall [87] | Analysis of non-specific stress markers | Gatherer-hunter | 224 | 0-5 years | Early-Late Prehistory, 4250 to 300 years BP | Central California, USA | North America | C. Temperate | - | Carbohydrates | Carbohydrates | - | - | - | **CF:** 3-5 years |
| Waterman [88] | Isotope Analysis | Agriculture | 81 | 28 juveniles aged 0-25 | Late Neolithic and Copper Age, 3500-1800 BC | Zambujal, Portugal | Europe & Central Asia | C. Temperate | terrestrial animal proteins, evidence of marine protein (though not a staple); C3 terrestrial proteins, | C3 plants; C3 terrestrial proteins, C 4 plants (eg. Millet potentially not consumed as staple crop, seaweed) | - | - | - | Statistically significant lower levels in Nitrogen isotope ratios for older juveniles, potentially a result of protein-restrictive child feeding practices/ different types of proteins consumed between adults and children or physiological processes related to skeletal development. | - |
| Waters-Rist [89] | Isotope Analysis | Gatherer-hunter-fisher | 49 | 0-10 yrs | Neolithic, 8800‐5200 BP | Cis-Baikal, Siberia | Europe & Central Asia | D. Continental | High in fat, potentially salmonoid fatty fish such as lenok (Brachymystax lenok) and the graylings (Thymallus sp.) or freshwater seals (Phoca sibirica). Comparative, non-fatty fish: non-fatty fishes include northern pike, cod and perch | - | A diet high in fat likely from salmonoid fish and freshwater seals | Premastication of salmonoid fish as well as giving infants chunks of seal fat to suck on - as this is what similar regions gatherer-hunter-fishers do today | - | Child diet is the same as adult diet. | **CF:** 0.75-3 years  **BC:** 2-4 years |
| Wilson [90] | Ethnography | gatherer-hunter-fisher-agriculture | 60 mothers | Mean age 40.26 years | Modern History, 2001 | North Rupununi, Guyana | Latin America & Caribbean | A. Tropical | - | manioc, sugar, bananas | sibe (manioc meal, water, and sugar mix), food from the mother’s plate, and ripe fruit such as bananas (Musa acuminata) | - | - | Complementary diet includes food from the "mother's plate" | **CF:** 57% of respondents introduced complementary foods at an average age of 3.9 months; 43% of respondents introduced complementary foods at an average of 8.2 months  **BC:** Mean of 19.32 months for mothers who  practiced EBF for less than 6 months and  could recall when they ceased all breastfeeding (n=25); Mean of 19.94 months for mothers who practiced EBF  for at least 6 months and could recall when  they ceased all breastfeeding (n=18) |
| Wilson [91] | Other | Gatherer-hunter-fisher-agriculture | 792 | 489 aged 0-9.99 years | Modern History, 1999-2002 | North Rupununi Region, Guyana | Latin America & Caribbean | A. Tropical | meat and dairy (5% of daily energy intake); fish provides an average of 49% of protein. | manioc (47% of daily energy intake); cereals/grains (12% of daily energy intake); nuts (11% of daily energy intake); fruits and vegetables (8% of daily energy intake); sugar (7% of daily energy intake) | - | - | Yes. Females are given more food than males. | - | **CF:** 6 ± 3 months  **BC:**20 ± 7 months |
| Wood [92] | Ethnography | Gatherer-hunter | 63 | Adult men aged 18+: (*Foods mentioned are provided to their wives and children, and are threfore part of the child diet.)* | Modern History, 2005-2009 | Lake Eyasi, Northern Tanzania | Sub-Saharan Africa | A. Tropical | honey, large game, small animals (dik-dik, impala, zebra, buffalo, eland, greater kudu) | fruits, tubers | - | - | - | - | - |
| Yi [93] | Isotope Analysis | Agriculture | 12 | 2 children aged 5 and 8 | Late Neolithic, 4500 BP | Chengdu Plain in Sichuan Province, China | East Asia & Pacific | D. Continental | animal protein from terrestrial and/or freshwater resources (pigs, dogs, deer, fish) | predominantly C3-based foods (rice); C4-foods (millet) | millet, animal protein | - | Yes. Females appear to have had more access to millet during weaning and children | - | **BC:** ~ 2.5 to 4 years |

**Supplementary Table 3.** **References for included studies**

| 1. Berbesque JC, Hoover KC. Frequency and developmental timing of linear enamel hypoplasia defects in Early Archaic Texan hunter-gatherers. *PeerJ* 2018;**6**:e4367.  2. Bird DW, Bliege Bird R. The Ethnoarchaeology of Juvenile Foragers: Shellfishing Strategies among Meriam Children. *J Anthropol Archaeol* 2000;**19**:461–76.  3. Bird DW, Bliege Bird R. Martu Children’s Hunting Strategies in the Western Desert, Australia. In: Hewlett BS, Lamb ME (eds.). *Hunter-Gatherer Childhoods: Evolutionary, Developmental, and Cultural Perspectives*. 1st ed. 2005, 129–46.  4. Bocherens H, Polet C, Toussaint M. Palaeodiet of Mesolithic and Neolithic populations of Meuse Basin (Belgium): evidence from stable isotopes. *J Archaeol Sci* 2007;**34**:10–27.  5. Boyette AH. Autonomy, cognitive development, and the socialisation of cooperation in foragers: Aka children’s views of sharing and caring. *Hunt Gatherer Res* 2019;**3**:475–500.  6. Budd C, Lillie M, Alpaslan-Roodenberg S *et al.* Stable isotope analysis of Neolithic and Chalcolithic populations from Aktopraklık, northern Anatolia. *J Archaeol Sci* 2013;**40**:860–7.  7. Bullington J. Deciduous dental microwear of prehistoric juveniles from the lower illinois River Valley. *Am J Phys Anthropol* 1991;**84**:59–73.  8. Castilla M, Carretero J-M, Gracia A *et al.* Evidence of rickets and/or scurvy in a complete Chalcolithic child skeleton from the El Portalón site (Sierra de Atapuerca, Spain). *J Anthropol Sci* 2014:257–71.  9. Chinique de Armas Y, Pestle W. Assessing the association between subsistence strategies and the timing of weaning among indigenous archaeological populations of the Caribbean. *Int J Osteoarchaeol* 2018;**28**:492–509.  10. Chinique de Armas Y, Roksandic M, Nikitović D *et al.* Isotopic reconstruction of the weaning process in the archaeological population of Canímar Abajo, Cuba: A Bayesian probability mixing model approach. Wiley AS (ed.). *PLOS ONE* 2017;**12**:e0176065.  11. Choy K, Jeon O-R, Fuller BT *et al.* Isotopic evidence of dietary variations and weaning practices in the Gaya cemetery at Yeanri, Gimhae, South Korea. *Am J Phys Anthropol* 2009:74–84.  12. Clayton F, Sealy J, Pfeiffer S. Weaning age among foragers at Matjes river rock shelter, South Africa, from stable nitrogen and carbon isotope analyses. *Am J Phys Anthropol* 2006;**129**:311–7.  13. Coltrain JB, Janetski JC. The stable and radio-isotope chemistry of southeastern Utah Basketmaker II burials: dietary analysis using the linear mixing model SISUS, age and sex patterning, geolocation and temporal patterning. *J Archaeol Sci* 2013;**40**:4711–30.  14. Cook DC, Buikstra JE. Health and differential survival in prehistoric populations: Prenatal dental defects. *Am J Phys Anthropol* 1979;**51**:649–64.  15. De Souza RG. Body size and growth: The significance of chronic malnutrition among the Casiguran Agta. *Ann Hum Biol* 2006;**33**:604–19.  16. Domínguez-Rodrigo M, Pickering TR, Diez-Martín F *et al.* Earliest Porotic Hyperostosis on a 1.5-Million-Year-Old Hominin, Olduvai Gorge, Tanzania. Smith FH (ed.). *PLoS ONE* 2012;**7**:e46414.  17. Early JD, Headland TN. *Population Dynamics of a Philippine Forest People: The San Ildefonso Agta*. Gainesville, FL: University Press of Florida, 1998.  18. Eerkens JW, Berget AG, Bartelink EJ. Estimating weaning and early childhood diet from serial micro-samples of dentin collagen. *J Archaeol Sci* 2011;**38**:3101–11.  19. Eerkens JW, Bartelink EJ. Sex-biased weaning and early childhood diet among middle holocene hunter-gatherers in Central California: Childhood Diet in Prehistoric Central California. *Am J Phys Anthropol* 2013;**152**:471–83.  20. Eerkens JW, Washburn E, Greenwald AM. Weaning and Early Childhood Diets at Two Early Period Sites: Implications for Parental Investment and Population Growth in Central California. *Calif Archaeol* 2017;**9**:199–222.  21. El-Najjar MY, Lozoff B, Ryan DJ. The paleoepidemiology of porotic hypertosis in the American Southwest: Radiological and ecological considerations. *Am J Roentgenol* 1975;**125**:918–24.  22. Eriksson G. Part-time farmers or hard-core sealers? Västerbjers studied by means of stable isotope analysis. *J Anthropol Archaeol* 2004;**23**:135–62.  23. Fernandes-Costa J. Transition from a hunter-gatherer to a settled lifestyle in the !Kung San: eftect on iron, folate, and vitamin B12 nutrition13. :9.  24. Fernández-Crespo T, Czermak A, Lee-Thorp JA *et al.* Infant and childhood diet at the passage tomb of Alto de la Huesera (north-central Iberia) from bone collagen and sequential dentine isotope composition. *Int J Osteoarchaeol* 2018;**28**:542–51.  25. Fernández-Crespo T, Ordoño J, Barandiarán I *et al.* The Bell Beaker multiple burial pit of La Atalayuela (La Rioja, Spain): stable isotope insights into diet, identity and mortuary practices in Chalcolithic Iberia. *Archaeol Anthropol Sci* 2019;**11**:3733–49.  26. Fontanals-Coll M, Eulàlia Subirà M, Díaz-Zorita Bonilla M *et al.* First insight into the Neolithic subsistence economy in the north-east Iberian Peninsula: paleodietary reconstruction through stable isotopes: The Neolithic Subsistence in the NE Iberia. *Am J Phys Anthropol* 2017;**162**:36–50.  27. Fouts HN, Hewlett BS, Lamb ME. Parent‐Offspring Weaning Conflicts among the Bofi Farmers and Foragers of Central Africa. *Curr Anthropol* 2005;**46**:29–50.  28. Fouts HN, Hewlett BS, Lamb ME. Weaning and the nature of early childhood interactions among bofi foragers in central Africa. *Hum Nat* 2001;**12**:27–46.  29. Fouts HN, Brookshire RA. Who feeds children? A child’s-eye-view of caregiver feeding patterns among the Aka foragers in Congo. *Soc Sci Med* 2009;**69**:285–92.  30. Gardner KS, Bartelink EJ, Martinez A *et al.* Breastfeeding and weaning practices of the ancestral Ohlone Indians of California: A case study using stable isotope analysis of bone collagen. *Int J Osteoarchaeol* 2018;**28**:523–34.  31. Garralda M, Maíllo‐Fernández J, Higham T *et al.* The Gravettian child mandible from El Castillo Cave (Puente Viesgo, Cantabria, Spain). *Am J Phys Anthropol* 2019;**170**:331–50.  32. Goodman AH, Armelagos GJ, Rose JC. The chronological distribution of enamel hypoplasias from prehistoric dickson mounds populations. *Am J Phys Anthropol* 1984;**65**:259–66.  33. Greenwald AM, Eerkens JW, Bartelink EJ. Stable isotope evidence of juvenile foraging in prehistoric Central California. *J Archaeol Sci Rep* 2016;**7**:146–54.  34. Halcrow SE, Harris NJ, Tayles N *et al.* From the mouths of babes: Dental caries in infants and children and the intensification of agriculture in mainland Southeast Asia. *Am J Phys Anthropol* 2013;**150**:409–20.  35. Han CS, Martin MA, Dichosa AEK *et al.* Salivary microbiomes of indigenous Tsimane mothers and infants are distinct despite frequent premastication. *PeerJ* 2016;**4**:e2660.  36. Hawkes K, O’Connell JF, Blurton Jones NG. Hunting and nuclear families: some lessons from the Hadza about men’s work. *Curr Anthropol* 2001;**42**:681–709.  37. Hawkes K, O’Connell JF, Blurton Jones NG. Hadza Women’s Time Allocation, Offspring Provisioning, and the Evolution of Long Postmenopausal Life Spans. *Curr Anthropol* 1997;**38**:551–77.  38. Hawkes K. Hadza Children’s Foraging: Juvenile Dependency, Social Arrangements, and Mobility among Hunter-Gatherers. *Curr Anthropol* 1995;**36**:688–700.  39. Herrscher E, Le Bras-Goude G. Southern French Neolithic populations: Isotopic evidence for regional specificities in environment and diet. *Am J Phys Anthropol* 2010:259–72.  40. Hirasawa A. Infant care among the sedentarized Baka hunter-gatherers in southeastern Cameroon. In: Hewlett BS, Lamb ME (eds.). *Hunter-Gatherer Childhoods: Evolutionary, Developmental, and Cultural Perspectives*. 1st ed. AldineTransaction, 2005, 365–84.  41. Howcroft R, Eriksson G, Lidén K. Infant feeding practices at the Pitted Ware Culture site of Ajvide, Gotland. *J Anthropol Archaeol* 2014;**34**:42–53.  42. Itahashi Y, Miyake Y, Maeda O *et al.* Preference for fish in a Neolithic hunter-gatherer community of the upper Tigris, elucidated by amino acid δ 15 N analysis. *J Archaeol Sci* 2017;**82**:40–9.  43. Itahashi Y, Erdal YS, Tekin H *et al.* Amino acid 15N analysis reveals change in the importance of freshwater resources between the hunter‐gatherer and farmer in the Neolithic upper Tigris. *Am J Phys Anthropol* 2019;**168**:676–86.  44. Ivey PK. Life-history theory perspectives on allocaretaking strategies among Efe foragers of the Ituri Forest of Zaïre. 1993.  45. Kamei N. Play among Baka Children in Cameroon. In: Hewlett BS, Lamb ME (eds.). *Hunter-Gatherer Childhoods: Evolutionary, Developmental, and Cultural Perspectives*. 1st ed. AldineTransaction, 2005, 343–59.  46. Katzenberg MA, Herring DA, Saunders SR. Weaning and infant mortality: Evaluating the skeletal evidence. *Yearb Phys Anthropol* **39**:23.  47. Keller M, Rott A, Hoke N *et al.* United in death-related by blood? Genetic and archeometric analyses of skeletal remains from the neolithic earthwork bruchsal-aue: Neolithic Multiple Burial: aDNA and Isotopes. *Am J Phys Anthropol* 2015;**157**:458–71.  48. Kinaston R, Bedford S, Richards M *et al.* Diet and Human Mobility from the Lapita to the Early Historic Period on Uripiv Island, Northeast Malakula, Vanuatu. Guatelli-Steinberg D (ed.). *PLoS ONE* 2014;**9**:e104071.  49. Kinaston RL, Buckley HR. Isotopic insights into diet and health at the site of Namu, Taumako Island, Southeast Solomon Islands. *Archaeol Anthropol Sci* 2017;**9**:1405–20.  50. King CL, Halcrow SE, Millard AR *et al.* Let’s talk about stress, baby! Infant-feeding practices and stress in the ancient Atacama desert, Northern Chile. *Am J Phys Anthropol* 2018;**166**:139–55.  51. King CL, Snoddy AM, Millard AR *et al.* A multifaceted approach towards interpreting early life experience and infant feeding practices in the ancient Atacama Desert, Northern Chile. *Int J Osteoarchaeol* 2018;**28**:599–612.  52. Kramer KL, Greaves RD. Synchrony between growth and reproductive patterns in human females: Early investment in growth among Pumé foragers. *Am J Phys Anthropol* 2010:235–44.  53. Lukacs JR. “From the mouth of a child”: dental attributes and health status during childhood in Mesolithic India. *Anthropol Sci* 2016;**124**:93–105.  54. Lynnerup N. The Thule Inuit Mummies From Greenland: The thule inuit mummies from greenland. *Anat Rec* 2015;**298**:1001–6.  55. Macchiarelli R. Prehistoric “fish-eaters” along the eastern Arabian coasts: Dental variation, morphology, and oral health in the Ra’s al-Hamra community (Qurum, Sultanate of Oman, 5th-4th millennia BC). *Am J Phys Anthropol* 1989;**78**:575–94.  56. Marsteller SJ, Zolotova N, Knudson KJ. Investigating economic specialization on the central Peruvian coast: A reconstruction of Late Intermediate Period Ychsma diet using stable isotopes: Marsteller et al. *Am J Phys Anthropol* 2017;**162**:300–17.  57. Martin MA, Garcia G, Kaplan HS *et al.* Conflict or congruence? Maternal and infant-centric factors associated with shorter exclusive breastfeeding durations among the Tsimane. *Soc Sci Med* 2016;**170**:9–17.  58. Meehan CL, Roulette JW. Early supplementary feeding among central African foragers and farmers: A biocultural approach. *Soc Sci Med* 2013;**96**:112–20.  59. Miller MJ, Dong Y, Pechenkina K *et al.* Raising girls and boys in early China: Stable isotope data reveal sex differences in weaning and childhood diets during the eastern Zhou era. *Am J Phys Anthropol* 2020;**172**:567–85.  60. Münster A, Knipper C, Oelze VM *et al.* 4000 years of human dietary evolution in central Germany, from the first farmers to the first elites. Halcrow SE (ed.). *PLOS ONE* 2018;**13**:e0194862.  61. Oelze VM, Siebert A, Nicklisch N *et al.* Early Neolithic diet and animal husbandry: stable isotope evidence from three Linearbandkeramik (LBK) sites in Central Germany. *J Archaeol Sci* 2011;**38**:270–9.  62. Ogrinc N, Budja M. Paleodietary reconstruction of a Neolithic population in Slovenia: A stable isotope approach. *Chem Geol* 2005;**218**:103–16.  63. Pearson JA, Haddow SD, Hillson SW *et al.* Stable carbon and nitrogen isotope analysis and dietary reconstruction through the life course at Neolithic Çatalhöyük, Turkey. *J Soc Archaeol* 2015;**15**:210–32.  64. Pearson JA, Hedges REM, Molleson TI *et al.* Exploring the relationship between weaning and infant mortality: An isotope case study from Aşıklı Höyük and Çayönü Tepesi. *Am J Phys Anthropol* 2010;**143**:448–57.  65. Scharlotta I, Goude G, Herrscher E *et al.* Shifting weaning practices in Early Neolithic Cis-Baikal, Siberia: New insights from stable isotope analysis of molar micro-samples. *Int J Osteoarchaeol* 2018;**28**:579–98.  66. Schniter E, Gurven M, Kaplan HS *et al.* Skill ontogeny among Tsimane forager-horticulturalists: Tsimane Skill Ontogeny. *Am J Phys Anthropol* 2015;**158**:3–18.  67. Schurr MR. Exploring ideas about isotopic variation in breastfeeding and weaning within and between populations: Case studies from the American midcontinent. *Int J Osteoarchaeol* 2018;**28**:479–91.  68. Schurr MR. Stable Nitrogen Isotopes as Evidence for the Age of Weaning at the Angel Site: A Comparison of Isotopic and Demographic Measures of Weaning Age. *J Archaeol Sci* 1997;**24**:919–27.  69. Sealy J, Pfeiffer S, Yates R *et al.* Hunter-Gatherer Child Burials from the Pakhuis Mountains, Western Cape: Growth, Diet and Burial Practices in the Late Holocene. *South Afr Archaeol Bull* 2000;**55**:32.  70. Sealy JC, Patrick MK, Morris AG *et al.* Diet and dental caries among later Stone Age inhabitants of the Cape Province, South Africa. *Am J Phys Anthropol* 1992;**88**:123–34.  71. Shack KW, Dewey KG, Grivetti LE. Effects of resettlement on the dietary intakes of mothers and children in lowland Papua new Guinea. *Ecol Food Nutr* 1990;**24**:55–70.  72. Shuler KA, Hodge SC, Danforth ME *et al.* In the shadow of Moundville: A bioarchaeological view of the transition to agriculture in the central Tombigbee valley of Alabama and Mississippi. *J Anthropol Archaeol* 2012;**31**:586–603.  73. Smith EK, Pestle WJ, Clarot A *et al.* Modeling Breastfeeding and Weaning Practices (BWP) on the Coast of Northern Chile’s Atacama Desert During the Formative Period. *J Isl Coast Archaeol* 2017;**12**:558–71.  74. Stantis C, Buckley HR, Kinaston RL *et al.* Isotopic evidence of human mobility and diet in a prehistoric/protohistoric Fijian coastal environment (c. 750-150 BP): Mobility and Diet in Fiji (c. 750-150 BP). *Am J Phys Anthropol* 2016;**159**:478–95.  75. Stefanović S, Petrović B, Porčić M *et al.* Bone spoons for prehistoric babies: Detection of human teeth marks on the Neolithic artefacts from the site Grad-Starčevo (Serbia). Biehl PF (ed.). *PLOS ONE* 2019;**14**:e0225713.  76. Szczepanek A, Belka Z, Jarosz P *et al.* Understanding Final Neolithic communities in south-eastern Poland: New insights on diet and mobility from isotopic data. Bondioli L (ed.). *PLOS ONE* 2018;**13**:e0207748.  77. Tessone A, García Guraieb S, Goñi RA *et al.* Isotopic evidence of weaning in hunter-gatherers from the late holocene in Lake Salitroso, Patagonia, Argentina: WEANING IN HUNTER-GATHERERS FROM PATAGONIA. *Am J Phys Anthropol* 2015;**158**:105–15.  78. Townsend PK. New Guinea sago gatherers: A study of demography in relation to subsistence. *Ecol Food Nutr* 1971;**1**:19–24.  79. Trancho GJ, Robledo B. Human skeletal remains from the Mesolithic site of Sheikh Mustafa (Central Sudan). An anthropometric and palaeodietary analysis. *Coplutum* 2003;**14**:401–8.  80. Tsutaya T, Ishida H, Yoneda M. Weaning age in an expanding population: stable carbon and nitrogen isotope analysis of infant feeding practices in the Okhotsk culture (5th-13th centuries AD) in Northern Japan: Weaning Age in the Okhotsk Culture. *Am J Phys Anthropol* 2015;**157**:544–55.  81. Tsutaya T, Yoneda M. Quantitative Reconstruction of Weaning Ages in Archaeological Human Populations Using Bone Collagen Nitrogen Isotope Ratios and Approximate Bayesian Computation. Konigsberg L (ed.). *PLoS ONE* 2013;**8**:e72327.  82. Tsutaya T, Shimomi A, Fujisawa S *et al.* Isotopic evidence of breastfeeding and weaning practices in a hunter–gatherer population during the Late/Final Jomon period in eastern Japan. *J Archaeol Sci* 2016;**76**:70–8.  83. Tucker B, Young AG. Growing up Mikea: Children’s Time Allocation and Tuber Foraging in Southwestern Madagascar. In: Hewlett BS, Lamb ME (eds.). *Hunter-Gatherer Childhoods: Evolutionary, Developmental, and Cultural Perspectives*. 1st ed. AldineTransaction, 2005, 147–71.  84. Ungar PS, Crittenden AN, Rose JC. Toddlers in Transition: Linear Enamel Hypoplasias in the Hadza of Tanzania: Hadza Linear Enamel Hypoplasias. *Int J Osteoarchaeol* 2017;**27**:638–49.  85. Valentin F, Bocherens H, Gratuze B *et al.* Dietary patterns during the late prehistoric/historic period in Cikobia island (Fiji): insights from stable isotopes and dental pathologies. *J Archaeol Sci* 2006;**33**:1396–410.  86. Veile A, Martin M, McAllister L *et al.* Modernization is associated with intensive breastfeeding patterns in the Bolivian Amazon. *Soc Sci Med* 2014;**100**:148–58.  87. Wall CE. Evidence of weaning stress and catch-up growth in the long bones of a Central California Amerindian sample. *Ann Hum Biol* 1991;**18**:9–22.  88. Waterman AJ, Tykot RH, Silva AM. Stable Isotope Analysis of Diet-based Social Differentiation at Late Prehistoric Collective Burials in South-Western Portugal: Social differentiation at collective burials in south-western Portugal. *Archaeometry* 2016;**58**:131–51.  89. Waters-Rist AL, Bazaliiskii VI, Weber AW *et al.* Infant and child diet in Neolithic hunter-fisher-gatherers from cis-baikal, Siberia: Intra-long bone stable nitrogen and carbon isotope ratios. *Am J Phys Anthropol* 2011;**146**:225–41.  90. Wilson W, Milner J, Bulkan J *et al.* Weaning practices of the Makushi of Guyana and their relationship to infant and child mortality: A preliminary assessment of international recommendations. *Am J Hum Biol* 2006;**18**:312–24.  91. Wilson WM, Bulkan J, Piperata BA *et al.* Nutritional status of Makushi Amerindian children and adolescents of Guyana. *Ann Hum Biol* 2011;**38**:615–29.  92. Wood BM, Marlowe FW. Household and Kin Provisioning by Hadza Men. *Hum Nat* 2013;**24**:280–317.  93. Yi B, Liu X, Yuan H *et al.* Dentin isotopic reconstruction of individual life histories reveals millet consumption during weaning and childhood at the Late Neolithic (4500 bp ) Gaoshan site in southwestern China. *Int J Osteoarchaeol* 2018;**28**:636–44. |
| --- |

**Supplementary Table 4.** Child evolutionary complementary feeding diets, by subsistence mode

| **FOOD GROUPS** | **GATHERER-HUNTER-FISHER^1^** | **EARLY AGRICULTURE^2^** |
| --- | --- | --- |
| *Fish and seafood* | Aquatic Mammals (2)  Finfish (13)  Mollusks & Crustaceans (3)  Roe (1)  Unspecified fish/seafood (1)  **N=20 (19.6%)** | Finfish (2)  Unspecified fish/seafood (2)  **N=4 (7.2%)** |
| *Meats, poultry, offal* | Birds (1)  Insects (4)  Mammals (2)  Other Mammals (2)  Reptiles (3)  Meat (animal source unspecified) (12)  **N=22 (21.6%)** | Unspecified animals (1)  Meat (animal source unspecified) (4)  **N=5 (9.1%)** |
| *Eggs* | **N=0 (0.0%)** | **N=0 (0.0%)** |
| *Milk and milk products* | Milk (1)  **N=1 (1.0%)** | Milk (1)  Unspecified Dairy products (1)  **N=2 (3.6%)** |
| *Pulses, legumes, nuts* | Nuts (5)  Acorns (3)  Unspecified Nuts (2)  Seeds (3)  Beans (2)  Peas (1)  Unspecified Legume (1)  **N=8 (7.8%)** | Seeds (1)  Beans (1)  Unspecified Pulse (1)  **N=2 (3.6%)** |
| *Vegetables* | Leafy Greens (2)  Mushrooms (1)  Unspecified vegetables (1)  **N=4 (3.9%)** | Unspecified vegetables (1)  Hot pepper sauce (2)  **N=3 (5.5%)** |
| *Fruits* | Baobab Fruit (1)  Plantains/Bananas (3)  Unspecified fruits (6)  **N=10 (9.8%)** | Plantains/Bananas (6)  Unspecified fruits (1)  **N=7 (12.7%)** |
| *Roots and tubers* | Cassava/Manioc (4)  Unspecified Root/Tuber (2)  Sago (1)  Sweet Potato (3)  Taro (3)  Yams (2)  **N=15 (14.7%)** | Cassava/Manioc (3)  Sweet Potato (1)  Taro (2)  Yams (2)  Unspecified Root/Tuber (1)  **N=9 (16.4%)** |
| *Cereals* | Maize/Corn (4)  Rice (3)  Sorghum (1)  Unspecified cereal (1)  **N=9 (8.8%)** | Maize/Corn (7)  Millet (1)  Rice (5)  Wheat (1)  Unspecified Cereal (1)  **N=15 (27.3%)** |
| *Oils and fats* | Animal fats (2)  Palm nuts/ palm oil (1)  **N=3 (2.9%)** | **N=0 (0.0%)** |
| *Sugar/honey* | Honey (2)  **N=2 (2.0%)** | Sugar/sugarcane (1)  Honey (1)  **N=2 (3.6%)** |
| *Other* | C3 plants (3)  Herbal teas (2)  Miscellaneous plants (2)  Unspecified foods (1)  **N=8 (7.8%)** | C3 plants (2)  C4 plants (1)  Herbal Teas (1)  Unspecified foods (2)  **N=6 (10.9%)** |

^1^The **gatherer-hunter-fisher** group is comprised of the *gatherer-hunter* and *gatherer-hunter-fisher* sub-categories.

^2^The **agriculture** group is comprised of the *gatherer-hunter-agriculture*, *gatherer-hunter-fisher-agriculture,* and *agriculture* sub-categories

**Supplementary Table 5.** Child evolutionary complementary feeding diets, by Köppen-Geiger climate zone

|  | 1. **Tropical (equatorial)** | 1. **Arid** | 1. **Temperate (warm/mild)** | 1. **Continental (snow)** | 1. **Polar (alpine)** |
| --- | --- | --- | --- | --- | --- |
| *Fish and seafood* | Finfish (4)  Mollusks & Crustaceans (1)  **N=5 (7.4%)** | Finfish (2)  Mollusk (1)  **N=3 (33.3%)** | Finfish (4)  Mollusks & Crustaceans (1)  Unspecified Seafood (2)  **N=7 (13.2%)** | Aquatic Mammals (2)  Finfish (4)  Roe (1)  Unspecified seafood (1)  **N=8 (50%)** | Finfish (1)  **N=1 (12.5%)** |
| *Meats, poultry, offal* | Birds (1)  Insects (3)  Meat (animal source unspecified) (8)  **N=12 (17.6%)** | Meat (animal source unspecified) (2)  **N=2 (22.2%)** | Mammals (1)   - Other Mammals (1)   Meat (animal source unspecified) (4)  Reptiles (3)  **N=8 (15.1%)** | **N=0 (0.0%)** | Meat (animal source unspecified) (2)  **N=2 (25.0%)** |
| *Eggs* | **N=0 (0.0%)** | **N=0 (0.0%)** | **N=0 (0.0%)** | **N=0 (0.0%)** | **N=0 (0.0%)** |
| *Milk and milk products* | **N=0 (0.0%)** | **N=0 (0.0%)** | Milk (2)  Unspecified Dairy Products (1)  **N=3 (5.7%)** | **N=0 (0.0%)** | **N=0 (0.0%)** |
| *Pulses, legumes, nuts* | Nuts (2)   - Unspecified nuts (2)   Seeds (1)   - Beans (1)   **N=3 (4.4%)** | **N=0 (0.0%)** | Nuts (3)   - Acorns (3)   Seeds (1)   - Beans (1)   Unspecified legume (1)  Unspecified pulse (1)  **N=6 (11.3%)** | **N=0 (0.0%)** | **N=0 (0.0%)** |
| *Vegetables* | Leafy Greens (2)  Mushrooms (1)  Hot pepper sauce (3)  Unspecified vegetables (1)  **N=7 (10.3%)** | **N=0 (0.0%)** | **N=0 (0.0%)** | **N=0 (0.0%)** | Unspecified vegetable (1)  **N=1 (12.5%)** |
| *Fruits* | Baobab fruit (1)  Plantains/Banana (9)  Unspecified fruits (3)  **N=13 (19.1%)** | **N=0 (0.0%)** | Unspecified Fruits (3)  **N=3 (5.7%)** | **N=0 (0.0%)** | Unspecified Fruits (1)    **N=1 (12.5%)** |
| *Roots and tubers* | Cassava/Manioc (4)  Sago (1)  Taro (1)  Yams (4)  Unspecified Root/Tuber (1)  **N=11 (16.2%)** | Unspecified Root/Tuber (1)  **N=1 (11.1%)** | Cassava/Manioc (3)  Sweet Potato (4)  Taro (4)  **N=11 (20.8%)** | Unspecified Root/Tuber (1)  **N=1 (6.3%)** | **N=0 (0.0%)** |
| *Cereals* | Maize (3)  Rice (8)  Sorghum (1)  **N=12 (17.6%)** | Maize (2)  **N=2 (22.2%)** | Maize (3)  Unspecified Cereal (1)  **N=4 (7.5%)** | Maize (3)  Millet (1)  Wheat (1)  **N=5 (31.3%)** | Unspecified Cereal (1)  **N=1 (12.5%)** |
| *Oils and fats* | Palm nuts/Palm oil (1)  **N=1 (1.5%)** | **N=0** | **N=0 (0.0%)** | Animal Fats (1)  **N=1 (6.3%)** | Animal Fats (1)  **N=1 (12.5%)** |
| *Honey and sugar* | Honey (3)  Sugar/Sugar cane (1)  **N=4 (5.9%)** | **N=0** | **N=0 (0.0%)** | **N=0 (0.0%)** | **N=0 (0.0%)** |
| *Other* | **N=0 (0.0%)** | C3 plants (1)  **N=1 (11.1%)** | C3 plants (3)  C4 plants (1)  Herbal Teas (3)  Miscellaneous plants (1)  Unspecified foods (3)  **N=11 (20.8%)** | C3 plants (1)  **N=1 (6.3%)** | Miscellaneous plants (1)  **N=1 (12.5%)** |
